# Involvement of endolysosome iron in HIV-1 gp120-, morphine-, and iron supplementation-induced disruption of the reactive species interactome and induction of neurotoxicity

**DOI:** 10.1080/13510002.2025.2546496

**Published:** 2025-08-21

**Authors:** Nirmal Kumar, Peter W. Halcrow, Darius N. K. Quansah, Braelyn Liang, Olimpia Meucci, Jonathan D. Geiger

**Affiliations:** a Department of Biomedical Sciences, University of North Dakota School of Medicine and Health Sciences, Grand Forks, ND, USA; b Department of Neuroscience and Cell Biology, Rutgers University Robert Wood Johnson Medical School, Piscataway, NJ, USA; c Department of Pharmacology and Physiology, Drexel University College of Medicine, Philadelphia, PA, USA

**Keywords:** HIV-1 gp120, morphine, endolysosome iron, reactive species interactome, reactive sulfur species, reactive nitrogen species, reactive oxygen species, reactive carbonyl species

## Abstract

Background: Iron continues to be linked to the pathogenesis of neurodegenerative disorders including HIV-1 associated neurocognitive disorders (HAND). People with HIV-1 who use opioids have a higher risk of developing HAND, and HIV-1 proteins and opioids disrupt endolysosome iron homeostasis, increase reactive oxygen species (ROS), and cause neural cell death. Endolysosomes are subcellular acidic organelles that regulate iron metabolism and redox homeostasis. HIV-1 gp120 and opioids induce endolysosome iron release, increasing cytosolic and in mitochondrial iron and ROS and inducing neurotoxicity. However, ROS represent only part of the reactive species interactome (RSI) and little is known about the extent to which HIV-1 proteins and opioids affect the RSI. Results: In SH-SY5Y and U87MG cells, HIV-1 gp120, morphine, and iron supplementation de-acidified endolysosomes, decreased endolysosome Fe^2+^ and H_2_S, and increased ROS, lipid peroxidation (LPO) and NO. These changes were accompanied by increased cytosolic and mitochondrial Fe^2+^, ROS, LPO, and NO, decreased H_2_S, and increased cell death. All effects were blocked by the endolysosome-specific iron chelator deferoxamine. Conclusion: Endolysosome iron dyshomeostasis induced by HIV-1 gp120, morphine and iron supplementation disrupts inter-organellar iron signaling and RSI homeostasis. Targeting endolysosome iron may mitigate neurotoxicity in HAND and other disorders associated with iron overload and redox imbalance.

## Introduction

HIV-associated neurocognitive disorder (HAND) affects about 50% of people living with HIV-1 (PLWH); clinical manifestations range from mild to severe and include deficits in cognition, memory, and motor function [1]. Although combined antiretroviral therapeutics (cART) are effective in suppressing HIV-1 load and improve to near normal levels life expectancy, the presence of latent HIV-1 reservoirs in PLWH appears to cause chronic neuroinflammatory effects attributed at least in part to pro-inflammatory HIV-1 proteins [2].

The pathogenesis of HAND is multifactorial and includes HIV-1 proteins, pro-inflammatory substances, and drugs of abuse [3]. Implicated in the neurotoxicity caused by these factors and to the pathogenesis of many diseases including HAND are elevated levels of reactive oxygen species (ROS) [4,5]. The HIV-1 coat protein gp120 and the opioid morphine are neurotoxic, increase levels of ROS [6,7], and PLWH ingesting opioids are at greater risk of oxidative stress-induced neuronal injury and developing worse cognitive outcomes of HAND [3]. However, targeting increases in ROS have been largely ineffective in preventing or reversing the pathogenesis of HAND and other neurodegenerative disorders [5,8–10] possibly due to the involvement of other reactive species.

The reactive species interactome (RSI) is a relatively new concept that in addition to ROS includes reactive sulfur species (RSS), reactive nitrogen species (RNS), and reactive carbonyl species (RCS); along with their downstream biological targets [8,9]. HIV-1 gp120 affects individual levels of reactive species in addition to ROS [6,11] but little is known what drives the overall dysregulation of the RSI including the redox-active transition metal ferrous iron (Fe^2+^).

Iron dyshomeostasis induces oxidative/nitrosative stress [12] and contributes to neurodegeneration in HAND [5,12–14]. Iron is essential for neuronal functions, but too much iron can promote oxidative stress through catalyzing free radical reactions that damage DNA and proteins thus resulting in neuronal death [12,13]. Accordingly, Fe^2+^ is compartmentalized, tightly regulated, and endolysosomes are acidic organelles that have been termed ‘master regulators of iron metabolism’ [15], and the dysregulation of Fe^2+^ may trigger RSI disruption and neurodegenerative disease progression.

Endolysosomes contain relatively high concentrations of readily releasable stores of Fe^2+^ [16,17]. The Fe^2+^ stores are sufficient to affect cytosolic and mitochondrial levels of Fe^2+^, redox signaling, and cellular events related to cell death [7,18,19]. The acidic lumen of endolysosomes is closely linked to iron homeostasis, and de-acidifying endolysosomes disrupts iron regulation [15,16,20]. Moreover, HIV-1 gp120 and opioids including morphine de-acidified endolysosomes and induced endolysosome Fe^2+^ release into the cytosol, and increased cytosolic and mitochondrial Fe^2+^ and ROS levels [7,19,21]. However, the role of endolysosome Fe^2+^ pool as well as the combined effects of gp120 and morphine on RSI components have not been reported.

Here, using SH-SY5Y human neuroblastoma and U87MG human astrocytoma cell lines we showed that endolysosome Fe^2+^ stores play an important role in regulating inter-organellar iron signaling, RSI homeostasis, and HIV-1 gp120-, morphine-, and iron supplementation-induced neurotoxicity (Figure 12). Targeting the RSI and iron stores may provide therapeutic benefits against HAND and other human diseases associated with endolysosome dysfunction, iron dyshomeostasis-induced toxicity, and increased levels of reactive species.

## Materials and methods

**Cell cultures:** SH-SY5Y human neuroblastoma and U87MG human astrocytoma cells were cultured in Dulbecco’s Modified Eagle Medium (1 x DMEM; Invitrogen, Carlsbad, U.S.A., Cat. #11995) containing 10% fetal bovine serum and 1% penicillin/streptomycin solution (Invitrogen, Carlsbad U.S.A., Cat. #15140122). Cells were grown in T75 (75 cm^2^) flasks and sub-cultured at 80–90% confluence. Cells were passaged every 3 to 4 days using 0.025% trypsin (Invitrogen, Carlsbad U.S.A., Cat. #25200056) and were not used past their 10th passage. Cells were maintained in a 5% CO_2_ humidified incubator at 37°C.

**Reagents and drug treatments:** Reagents were purchased as follows; recombinant HIV-1 IIIB gp120 (ImmunoDX, Woburn, MA, U.S.A., Cat. #1001), morphine sulfate and deferoxamine mesylate salt (Sigma-Aldrich, St. Louis, U.S.A., Cat. #M8777 and Cat. #D9533), and ferric ammonium citrate (Fisher Scientific, Waltham, MA, U.S.A., Cat. #I72-500). Stock solutions of HIV-1 gp120 (1 μM) and morphine (5 mM) were prepared in ultrapure distilled water (Invitrogen, Waltham, MA, U.S.A., Cat. #10977015) and freshly diluted to working concentrations immediately prior to use. Distilled water was used as vehicle control unless otherwise specified. The final concentrations of morphine (1 μM) and HIV-1 gp120 (500 pM or ∼60 ng/mL) were within the ranges measured in humans; patients chronically treated for pain had plasma levels of morphine ranging from 10 nM to 1 μM [22,23] and serum concentrations of HIV-1 gp120 ranged from 120 to 960 ng/mL [24]. Additionally, we examined concentration-dependent effects of gp120, morphine, and ferric ammonium citrate (FAC) for their ability to increase cytosolic ROS and found that gp120, morphine, and FAC increased cytosolic ROS levels within 1 h of treatment (Supplementary Figure S12). Based on the literature and our concentration-dependent studies, concentrations used were as follows; gp120 (500 pM), morphine (1 μM), and FAC (50 μM). To determine early upstream effects of endolysosome dysfunction on mitochondria and neural cell death, we used 1 h incubations unless otherwise indicated.

**Endolysosome pH measurements:** LysoSensor Green DND-189 (Invitrogen, Waltham, MA, U.S.A., Cat. #L7535) was used to monitor semi-quantitative changes in endolysosome pH. SH-SY5Y cells were seeded in 35 mm^2^ dishes with thin glass bottoms coated with poly-D-lysine (Fisher Scientific, Waltham, MA, U.S.A., Cat. #NC0969321), and plated cells were incubated at 37°C and 5% CO_2_. After drug treatments for 1 h, cells were stained for 7 min with 40 nM LysoSensor Green DND-189 and 1 μg/ml Hoechst 33342 (ThermoFisher, Waltham, MA, U.S.A., Cat. #62249). Cells were washed three times with PBS and resuspended in live cell imaging solution (Invitrogen, Waltham, MA, U.S.A., Cat. #A14291DJ). Fluorescence was excited at 488 nm and emission was collected at 520 nm using our Andor Dragonfly 200 spinning disk confocal microscope (Oxford Instruments, Concord, U.S.A.) equipped with a 63x oil-immersion objective. The mean fluorescence intensity (MFI) was determined with Imaris software version 9.9.1. For quantitative measurements of endolysosomal pH, the ratiometric probe LysoSensor Yellow/Blue DND-160 (Invitrogen, Waltham, MA, U.S.A., Cat. #L7545) was used. SH-SY5Y cells were grown overnight in 96-well black/clear bottom plates (Invitrogen, Waltham, MA, U.S.A., Cat. #165305). Cells were treated with different drugs for 30 min or first preincubated with DFO for 30 min. After drug treatment, the culture medium was removed and cells were incubated in imaging buffer containing 5 μM LysoSensor Yellow/Blue DND-160 for 4 min at room temperature in the dark. The imaging solution contained 140 mM NaCl, 2.5 mM KCl, 1.8 mM CaCl_2_, 1 mM MgCl_2_, and 20 mM HEPES (pH 7.4). Immediately after staining, cells were rinsed three times with imaging buffer to remove the extracellular dye. Fluorescence intensity was measured using a microplate reader (BioTek Synergy H1); dual excitation was at 340 nm (F_340_ nm) and 380 nm (F_380_ nm), and emission was collected at 527 nm. Relative endolysosomal pH was determined from the F_340_ nm/F_380_ nm ratio. To quantify endolysosome pH values, calibrations were performed using a series of pH calibration buffers (pH 4.0, 4.5, 5.0, 5.5, 6.0, and 6.5) in a calibration solution containing 5 mM NaCl, 115 mM KCl, 1.2 mM MgSO_4_, 10 mM glucose, and 25 mM HEPES supplemented with 10 μM nigericin and 10 μM valinomycin. Cells were incubated with calibration buffers at room temperature for 10 min after staining with the ratiometric probes and the fluorescence ratios were plotted against the pH. Endolysosome pH was calculated by fitting their fluorescent ratios to the pH standard curve using GraphPad Prism software. Using the formula pH = -log [H^+^], we calculated percent changes in intraluminal H^+^ concentrations.

**Endolysosome Fe^2+^, ROS, and LPO measurements:** SH-SY5Y cells were added to 35 mm^2^ culture dishes and incubated overnight at 37°C and 5% CO_2_. After treatments and 1 h incubation at 37°C, cells were incubated with one of the following probes; 10 μM FeRhonox^TM^-1 (endolysosome Fe^2+^, Goryo, Chemical, Cat. #SCT030) for 25 min; 5 μM PF-H_2_TMRos (ROS; Invitrogen, Cat. #R14060) for 30 min; 5 μM CellROX^TM^ Deep Red reagent (ROS; Invitrogen, Cat. #C10422) for 30 min; or 10 μM BODIPY^TM^ 581/591 C11 (LPO, Invitrogen, Cat. #D3861) for 25 min. During the final 7 min of staining, 50 nM of LysoTracker^TM^ green DND-26 (504/511 nm, Invitrogen, Cat. #L7526) or LysoTracker deep red (647/668 nm, Invitrogen, Cat. #L12492) was added to label endolysosomes along with 1 μg/ml Hoechst 33342 (405/460 nm) to label nuclei. Cells were washed three times with PBS and resuspended in live cell imaging solution. Cells were imaged with our spinning disk confocal microscope with the following settings; 540/590 nm (excitation/emission) for FeRhonox-1, 540/600 nm for PF-H_2_TMRos, and 644/665 nm for CellROX^TM^ Deep Red reagent. BODIPY C11 fluorescence was visualized using dual-channel acquisition; the oxidized (green) form with 488 nm excitation and ∼510–520 nm emission, and the non-oxidized (red) form with 561 nm excitation and ∼590 emission. CellROX^TM^ Deep Red reagent exhibits a strong fluorescent signal upon oxidation by hydroxyl radical (^•^OH), superoxide (O_2_^• -^), and hydrogen peroxide (H_2_O_2_) [25]. PF-H_2_TMRos detects lysosomal ^•^OH and other ROS [26]. MFI was determined using Imaris software version 9.9.1.

**Endolysosome and mitochondrial NO and H_2_S, and extracellular H_2_S measurement:** SH-SY5Y cells were added to 35 mm^2^ culture dishes and incubated overnight at 37°C and 5% CO_2_. Following 1 h treatments, media was aspirated and cells were washed two times with PBS before incubation at 37°C for 25 min with 10 μM DAF-FM DA (Invitrogen, Cat. #D23844) in phenol red-free DMEM. Cells were then washed two times with PBS, resuspended in 1.5 mL phenol red-free DMEM, and incubated for an additional 15 min at 37°C to promote intracellular de-esterification. For H_2_S measurement, cells were incubated for 20 min at 37°C with 10 μM of the H_2_S-specific fluorescent probe P3 (Sigma-Aldrich, Cat. #5.34329). Cells were subsequently stained for 7–10 min at 37°C with 50 nM LysoTracker deep red (647/668 nm) to label endolysosomes, 50 nM MitoTracker deep red (644/665 nm; Invitrogen, Cat. #M22426) for mitochondria, and 1 μg/mL Hoechst 33342 for nuclei. After incubation, cells were washed three times, resuspended in 1.5 mL of live-cell imaging solution, and imaged using our spinning disk confocal microscope equipped with 63x oil-immersion objective. DAF-FM DA and P3 fluorescence were detected using 488 nm excitation and 520 nm emission, and MFI was determined using Imaris software (version 9.9.1).

For extracellular H_2_S measurements, cells were cultured in 24-well plates (Thermo Scientific, Waltham, MA, U.S.A., Cat. #142475) and SF7-AM (0.3 μM, Cayman Chemicals, Cat. #1416872-50-8) was added to the cell culture media together with various treatments. After 1 h incubation at 5% CO_2_ and 37°C, 100 μL of media was transferred to a 96-well clear-bottom black-sided plate and fluorescence was measured at an excitation of 480 nm and emission of 520 nm with a microplate reader. P3 and SF7-AM probes have been previously validated as selective for H_2_S over other biologically relevant thiols [27–29]. For validation and as a positive control, cells were treated with exogenous NaHS and incubated in serum-free media for 2 h to induce endogenous H_2_S production. Aminooxyacetic acid (AOAA), a CBS inhibitor, was used to suppress endogenous H_2_S production.

**Cytosolic Fe^2+^, ROS, and H_2_S measurements:** Cytosolic Fe^2+^, ROS, H_2_O_2_ and H_2_S levels were measured using Phen Green^TM^ FL, diacetate (PGFL-DA; Invitrogen, Cat. #P14313) for Fe^2+^, CM-H_2_DCFDA (Invitrogen, Cat. #C6827) for ROS, H_2_O_2_ assay kit (Abcam, Boston, MA, U.S.A., Cat. #ab138874) for H_2_O_2_, and SF7-AM for H_2_S. SH-SY5Y Cells (3–6 × 10^5^ cells/well) were added to 24-well plates and incubated at 37°C and 5% CO_2_ overnight prior to being taken for experimentation. Cells were preincubated with 50 μM DFO for 1 h prior to the addition of various treatments and incubations for an additional 1 h. Cells were washed three times with PBS and incubated with 10 µM CM-H_2_DCFDA in PBS for 20 min or with H_2_O_2_ assay kit solution and/or SF7-AM (0.3 μM) in culture media for 25 min in a 37°C and 5% CO_2_ incubator. For cytosolic Fe^2+^ detection, cells were first stained with 10 µM PGFL-DA in culture media for 20 min, followed by 1 h preincubation with DFO, then treated for 1 h as above. After incubations, cells were washed three times with PBS, cells were collected and resuspended in 500–600 μL PBS for flow cytometry. Cells treated with 50 μM H_2_O_2_ used as positive controls for ROS and cells incubated in FBS-free media and treated with 1 mM NaHS were used as H_2_S-positive controls. Mean fluorescence intensity (MFI) was measured using the BL1 channel (488 nm excitation, 530 nm emission) on our Attune NxT flow cytometer (ThermoFisher, Waltham, U.S.A.). Flow cytometry was performed by acquiring 10,000 events per sample. Each sample was measured at least three times, and the average MFI from these measurements was used for analyses.

**Mitochondrial Fe^2+^, superoxide (O_2_^• −^), hydroxy radical (^•^OH), hydrogen peroxide (H_2_O_2_) and lipid peroxidation (LPO) measurements:** Mitochondrial Fe^2+^, O_2_^• −*,* •^OH, H_2_O_2_ and LPO levels were measured using rhodamine B 4-[(2,2’-bipyridin-4-yl) aminocarbonyl] benzyl ester (RDA) (Guidechem, Milwaukee, U.S.A., Cat. #952228-30-7), MitoSOX (Invitrogen, Cat. #M360089), mitochondrial ^•^OH detection assay kit (Abcam, Cat. #ab219931), mitochondrial H_2_O_2_-specific probe, MitoPY1 (Tocris, Bristol, U.K., Cat. #4428), and a mitochondrial LPO-specific probe MitoPerOX BODIPY (Abcam, Cat. #ab146820), respectively. SH-SY5Y cells (3× 10^5^ cells/well) were added to 24-well plates and incubated at 37°C and 5% CO_2_ overnight prior to being taken for experimentation. Cells were preincubated with 50 μM DFO for 1 h, then treated with gp120 and/or morphine for an additional 1 h. Following treatment, cells were incubated at 37°C in a humidified 5% CO_2_ incubator with one of the following mitochondrial probes; 100 nM RDA (20 min), 2.5 µM of MitoSOX^TM^ (20 min), mitochondrial ^•^OH detection solution (45 min), 10 μM MitoPY1 (20 min), or 200 nM MitoPerOX BODIPY (25 min). Cells were rinsed three times with PBS, resuspended in 500 or 600 μL PBS, and collected into 1.5 mL Eppendorf tubes. RDA fluorescence was measured by flow cytometry using 562 nm excitation and ∼598 nm emission. MitoSOX and ^•^OH fluorescence was detected using the BL2 channel, MitoPYI fluorescence was measured using the BL1 channel, and MitoPerOX BODIPY fluorescence was measured using BL1 and BL2 channels. Flow cytometry was performed by acquiring 10,000 events per sample. Each sample was measured at least three times, and the average MFI from these measurements was used for analyses.

**Cell death:** SH-SY5Y cells (about 60,000/well) were added in 24 well plates and incubated for 24 h at 37°C and 5% CO_2_. Following the addition of fresh DMEM media, treatments were applied for 24 h. DFO (50 μM) was added 1 h before addition of gp120 (500 pM) and morphine (1 μM) and incubation for 24 h. Following removal of the media, cells were collected by aspiration using 0.05% trypsin (ThermoFisher Scientific, Cat. #25300054) and added to the collected media. Cells were spun down at 400 x g for 5 min and washed twice with PBS to remove residual media. Cells were stained with propidium iodide (PI, 3 µM, BD Biosciences, Cat. #556463) at room temperature for 15 min in the dark, and fluorescence was measured by flow cytometry at an excitation of 535 nm and emission of 617 nm. For each sample, 10,000 events were acquired in triplicate, and the average MFI from these measurements was used for analyses.

**Statistics and reproducibility:** All experiments were repeated multiple times to ensure reproducibility. Statistical parameters including *p* values, sample sizes (n), and biological replicates were reported in the Results section, in Figures, and in Figure Legends. Comparison of two groups was performed using two-tailed unpaired Student’s *t*-test. One-way analysis of variance (ANOVA) was used to compare data from multiple treatment groups and Tukey’s multiple comparison was used to determine statistically significant differences. Normality was tested using Shapiro-Wilk’s test and Kolmogorov-Smirnov’s test. A level of significance *p* < 0.05 was used. The presence of outliers was tested using ROUT method (Q = 1%) in GraphPad Prism. GraphPad Prism software (version 9.4.0 to 9.5.1) was used to perform all statistical analyses.

## Results

**Figure 1.**
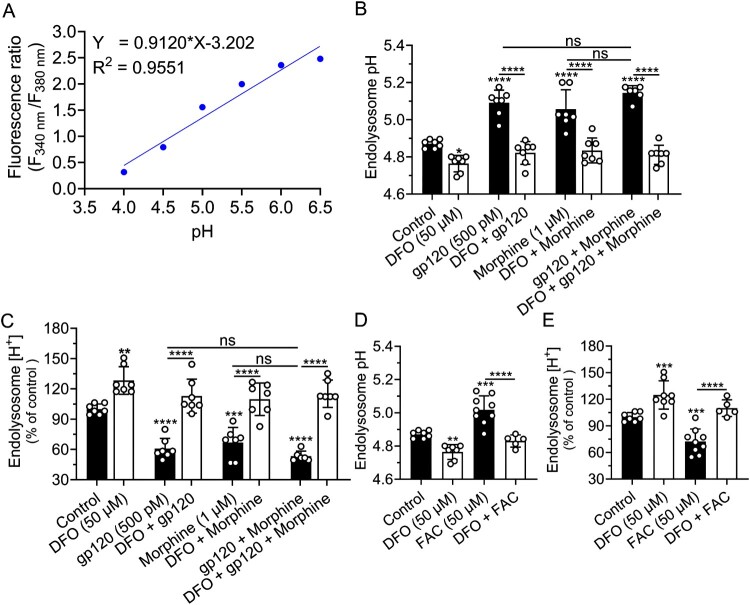
DFO blocked gp120-, morphine-, and iron supplementation-induced endolysosome de-acidification. (A) A standard curve for endolysosome pH calibration was determined using LysoSensor Yellow/Blue DND-160 and a microplate reader. (B–E) Quantitative changes in endolysosome pH and proton (H^+^) concentration in SH-SY5Y cells pretreated for 30 min with DFO (50 μM) or water (control) and then treated for 30 min with gp120 (500 pM), morphine (1 μM), gp120 plus morphine, and FAC (50 μM). Data were presented as means ± SD, with individual data points (*n* = 5–10) included on each bar. Each data point (*n* = 5–10) represents mean pH measurements from cells in 96-well plates. One-way ANOVA with Tukey’s multiple comparison test was used for statistical analyses. ns = non-significant, *p < 0.05, **p < 0.01, ***p < 0.001, ****p < 0.0001.

Deferoxamine (DFO) blocked gp120-, morphine-, and iron supplementation-induced endolysosome de-acidification*.* Quantitative analysis of LysoSensor Yellow/Blue DND-160 fluorescence staining at various pH levels (Figure 1(A)) showed that pre-incubation of SH-SY5Y cells for 30 min with DFO (50 μM) significantly (*p* < 0.05) acidified endolysosomes and significantly (*p* < 0.0001) blocked gp120-, morphine-, gp120 plus morphine, and FAC-induced endolysosome de-acidification (Figure 1(B–E)). The endolysosome pH changes corresponded to decreases in intraluminal H^+^ concentrations of 39% for gp120, 33% for morphine, 47% for gp120 plus morphine, and 28% for FAC (Figure 1(C,E)). DFO induced 25–28% increases in H^+^ and blocked gp120-, morphine-, gp120 plus morphine, and FAC-induced decreases in endolysosome H^+^ (Figure 1(C,E)). Similar results were observed with the LysoSensor DND-189 dye (Supplementary Figure S1A-D). Heat-inactivated gp120 (Hi-gp120, 500 pM) did not significantly affect endolysosome pH (Supplementary Figure S1E-H).

**Figure 2.**
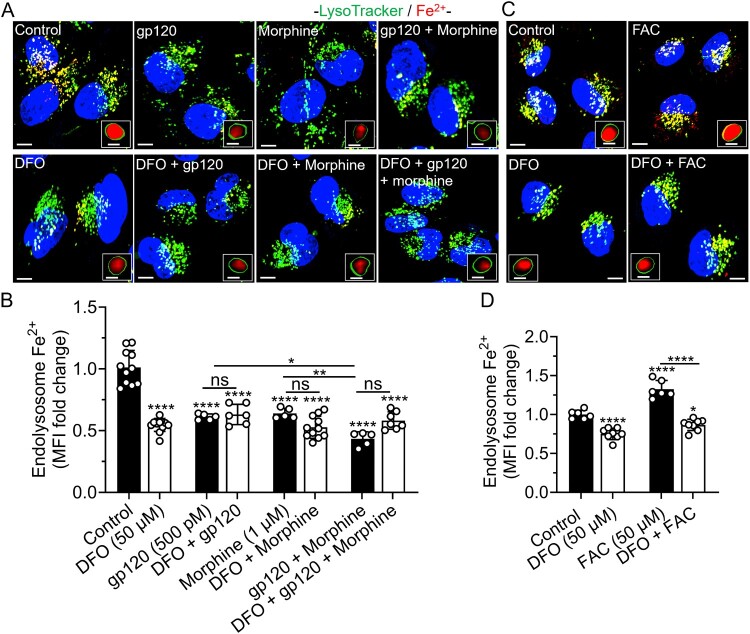
DFO blocked FAC-induced increases in endolysosome Fe^2+^ but not gp120-, morphine-, and gp120 plus morphine-induced decreases in endolysosome Fe^2+^. (A, C) Representative images of SH-SY5Y cells pretreated for 1 h with DFO (50 μM) or water (control), and then treated for 1 h with gp120 (500 pM), morphine (1 μM), gp120 plus morphine, and FAC (50 μM) prior to staining for Fe^2+^ (FeRhoNox-1, red), endolysosomes (LysoTracker Green, green) and nuclei (Hoechst 33342, blue). Scale bars = 10 μm. Boxed inserts are magnifications of composite endolysosomes (green) containing Fe^2+^ (red). Scale bars = 1 μm. (B, D) Semi quantitative changes in endolysosome Fe^2+^ were determined from images using Imaris 3D software and data were represented as fold-changes of mean fluorescence intensity (MFI) of FeRhoNox-1 staining of LysoTracker-positive endolysosomes. Means ± SD, with individual data points (*n* = 5–11) were shown on each bar. Each data point (n) represents MFI of FeRhonox-1 staining inside LysoTracker-positive endolysosomes from multiple cells within a field of view. A minimum of 100 cells per condition from two biological replicates were analyzed. One-way ANOVA with Tukey’s multiple comparison test was used for statistical analyses. ns = non-significant, *p < 0.05, **p < 0.01, ****p < 0.0001.

DFO blocked FAC-induced increases in endolysosome Fe^2+^ but not gp120-, morphine-, and gp120 plus morphine-induced decreases in endolysosome Fe^2+^. gp120 (500 pM), morphine (1 μM), and gp120 plus morphine decreased FeRhoNox-1 fluorescence staining in SH-SY5Y cells for Fe^2+^ (Figure 2(A,B)). Pretreatment with DFO (50 μM) significantly (*p* < 0.0001) decreased FeRhoNox-1 fluorescence staining for Fe^2+^ (Figure 2(B)), blocked FAC (50 μM) induced increases in FeRhoNox-1 fluorescence staining for Fe^2+^ (Figure 2(C,D)), but did not significantly affect gp120- (500 pM), morphine- (1 μM), and gp120 plus morphine-induced decreases in FeRhoNox-1 fluorescence staining for Fe^2+^ (Figure 2(A,B)). Hi-gp120 (500 pM) did not significantly affect FeRhoNox-1 fluorescence staining for Fe^2+^ (Supplementary Figure S2A, B).

**Figure 3.**
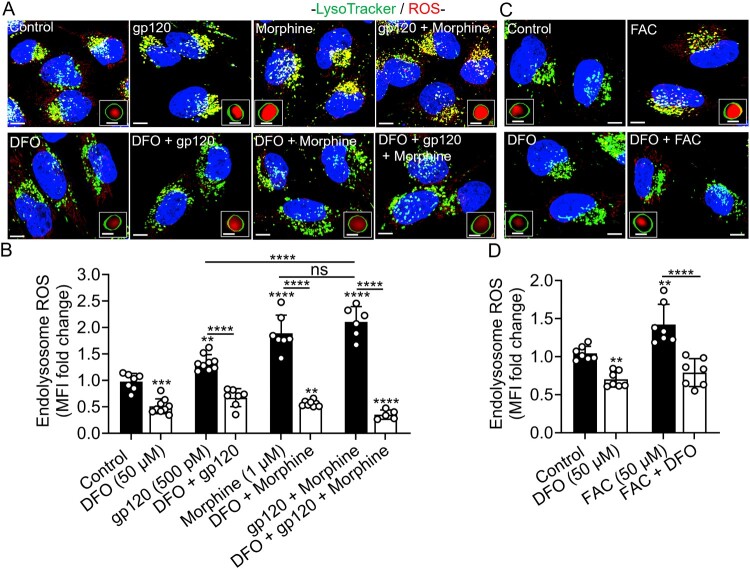
DFO decreased endolysosome reactive oxygen species (ROS), and blocked gp120-, morphine- and iron supplementation-induced increases in endolysosome ROS. (A, C) Representative images of SH-SY5Y cells pretreated for 1 h with DFO (50 μM) or water (control) and then treated for 1 h with gp120 (500 pM), morphine (1 μM), gp120 plus morphine, and FAC (50 μM) prior to staining ROS with PF-H2TMRos (red), endolysosomes with LysoTracker Green (green), and nuclei with Hoechst 33342 (blue). Scale bars = 10 μm. Boxed inserts are magnifications of single composite endolysosomes containing ROS. Scale bars = 1 μm. (B, D) Semi-quantitative changes in endolysosome ROS were determined using Imaris 3D software and data were represented as fold-changes of mean fluorescence intensity (MFI) of PF H2TMRos in LysoTracker-positive endolysosomes. Data were presented as means ± SD, with individual data points (*n* = 5–10) shown on each bar. Each data point represents MFI of PF-H2TMRos staining inside LysoTracker-positive endolysosomes from multiple cells within a field of view. A minimum of 100 cells per condition from two biological replicates were analyzed. One-way ANOVA with Tukey’s multiple comparison test was used for statistical analyses. ns = non-significant, **p < 0.01, ***p < 0.001, ****p < 0.0001.

**Figure 4.**
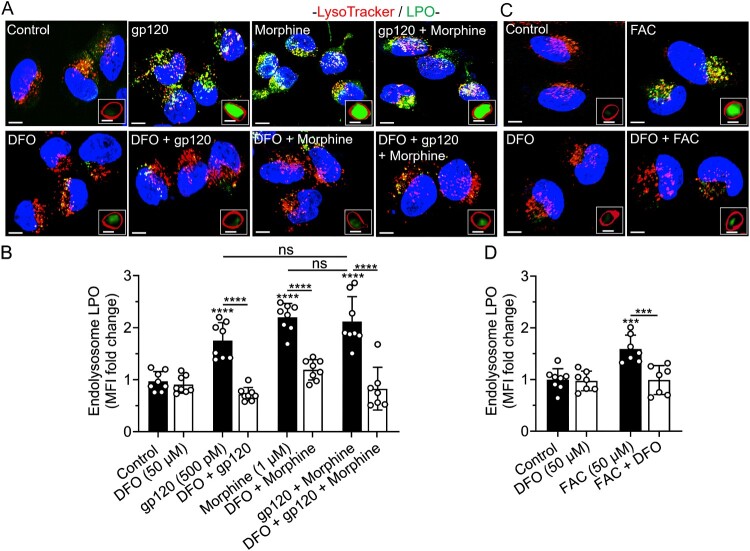
DFO blocked gp120-, morphine-, and iron supplementation-induced increases in endolysosome lipid peroxidation. (A, C) Representative images of SH-SY5Y cells pretreated for 1 h with DFO (50 μM) or water (control), and then treated for 1 h with gp120 (500 pM), morphine (1 μM), gp120 plus morphine, and FAC (50 μM) before staining for lipid peroxidation (LPO) with Bodipy 581/591 C11 (green), endolysosomes with LysoTracker Far Red (red), and nuclei with Hoechst 33342 (blue). Scale bars = 10 μm. Boxed inserts are magnifications of single composite endolysosomes containing oxidized Bodipy C11. Scale bars = 1 μm. (B, D) Images were quantified using Imaris 3D software and data were represented as fold-changes of mean fluorescence intensity (MFI) of oxidized Bodipy C11 in LysoTracker-positive endolysosomes. Means ± SD, with individual data points (*n* = 7–8) were shown on each bar. Each data point (n) represents MFI of oxidized Bodipy C11 staining in LysoTracker-positive endolysosomes from multiple cells within a field of view (per image). A minimum of 66 cells from three biological replicates were analyzed. One way ANOVA with Tukey’s multiple comparison test was used for statistical analyses. ns = non-significant, ***p < 0.001, ****p < 0.0001.

**Figure 5.**
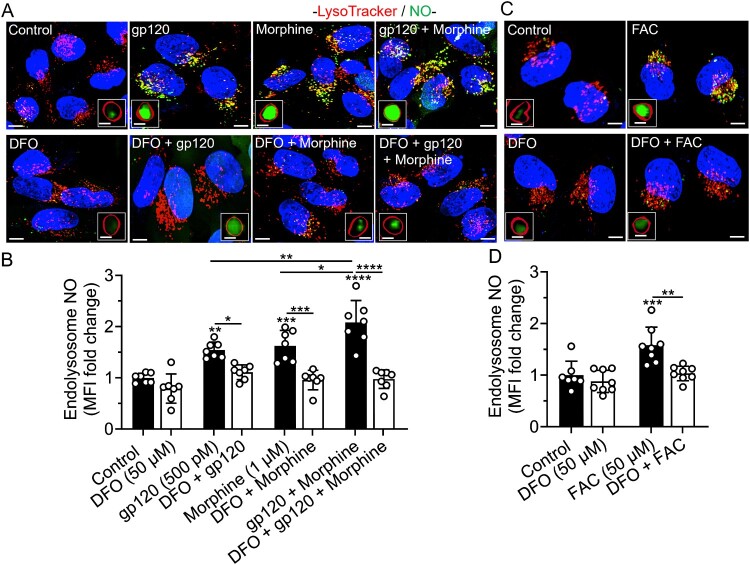
DFO blocked gp120-, morphine-, and iron supplementation-induced increases in endolysosome nitric oxide. (A, C) Representative confocal images of SH-SY5Y cells pretreated for 1 h with DFO (50 μM) or water (control) prior to treatment for 1 h with gp120 (500 pM), morphine (1 μM), gp120 plus morphine, and FAC (50 μM) and then staining for nitric oxide (NO) with DAF-FM DA (green), endolysosomes with LysoTracker Red (red) and nuclei with Hoechst 33342 (blue). Scale bars = 10 μm. Boxed inserts are magnifications of single composite endolysosomes containing NO. Scale bars = 1 μm. (B, D) Semi-quantitative changes in endolysosome NO were determined using Imaris 3D software and data were represented as fold changes in mean fluorescence intensity (MFI) of DAF-FM DA staining in LysoTracker positive endolysosomes. Bars indicate mean ± SD; individual data points (*n* = 6–8) were included on each bar. Each data point (n) represents MFI of DAF-FM DA staining in LysoTracker-positive endolysosomes from multiple cells. A minimum of 133 cells per condition from two biological replicates were analyzed. One-way ANOVA with Tukey’s multiple comparison test was used for analysis. *p < 0.05, **p < 0.01, ***p < 0.001, ****p < 0.0001.

DFO blocked gp120-, morphine, gp120 plus morphine-, and iron supplementation-induced increases in endolysosome ROS, LPO and NO levels. Fluorescence imaging using PF-H_2_TMRos (ROS), BODIPY C11 (lipid peroxidation), and DAF-FM DA (NO) showed that gp120 (500 pM), morphine (1 μM), gp120 plus morphine, and FAC (50 μM) significantly increased endolysosome ROS, LPO, and NO levels (Figures 3–5(A–D)). ROS levels in SH-SY5Y cells treated with gp120 plus morphine were significantly (*p* < 0.0001) higher than those induced by gp120 alone, but not morphine alone (Figure 3(B)). gp120 plus morphine had no significant additive effects on LPO levels (Figure 4(A,B)), but it did induce a significant increase in NO levels compared with gp120 or morphine alone (Figure 5(A,B)). ROS levels were significantly increased by H_2_O_2_ (150 μM; *p* < 0.0069) and significantly reduced by the antioxidant N-acetyl cysteine (NAC, 5 mM; *p* < 0.0095) (Supplementary Figure S3A, B). RSL3 (10 μM), used as positive control, significantly (*p* < 0.0001) increased endolysosome LPO levels. In contrast, Hi-gp120 (500 pM) did not induce significant increases in ROS, LPO, or NO levels (Supplementary Figure S3–5, A, B), confirming that the observed effects were specific to native gp120. Pre-treatment with DFO (50 μM) significantly blocked the gp120-, morphine-, gp120 plus morphine- and FAC-induced increases in endolysosome ROS, LPO, and NO levels (Figure 3–5), indicating that these responses are mediated by endolysosome iron-dependent mechanisms.

**Figure 6.**
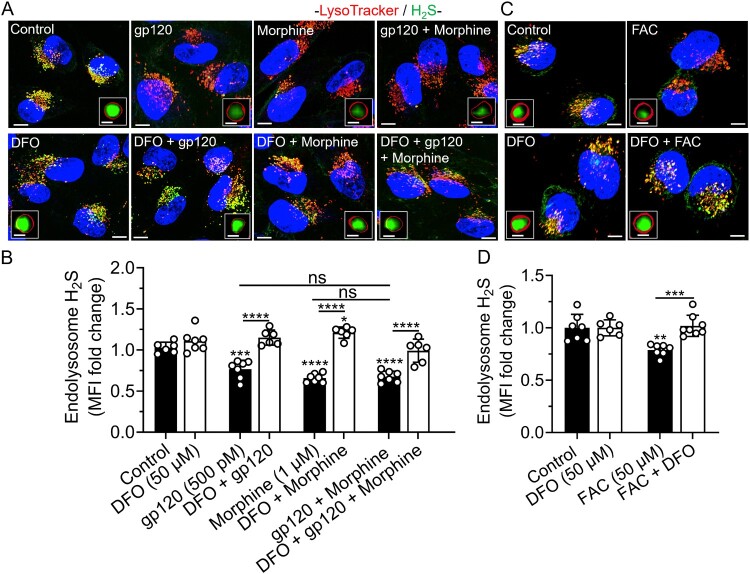
DFO blocked gp120-, morphine-, and iron supplementation-induced decreases in endolysosome H_2_S. (A, C) Representative confocal images of SH-SY5Y cells pretreated for 1 h with DFO (50 μM) or water (control) and then treated for 1 h with gp120 (500 pM), morphine (1 μM), gp120 plus morphine, and FAC (50 μM) before staining for H_2_S with P3 probe (green), endolysosomes with LysoTracker Red (red) and nuclei with Hoechst 33342 (blue). Scale bars = 10 μm. Boxed inserts are magnifications of single composite endolysosomes containing H_2_S. Scale bars = 1 μm. (B, D) Semi-quantitative changes in endolysosome H_2_S were determined using Imaris 3D software and data were represented as fold changes in mean fluorescence intensity (MFI fold change) of P3 in LysoTracker-positive endolysosomes. Data were shown as mean ± SD, with individual data points (*n* = 6–8) included on each bar. Each data point (n) represents MFI of P3 staining inside LysoTracker-positive endolysosomes from multiple cells. A minimum of 100 cells per condition from two biological replicates were analyzed. One-way ANOVA with Tukey’s multiple comparison test was used for statistical analyses. ns = non-significant, *p < 0.05, **p < 0.01, ***p < 0.001, ****p < 0.0001.

DFO blocked gp120-, morphine-, gp120 plus morphine-, and iron supplementation-induced decreases in endolysosome hydrogen sulfide (H_2_S). Using the H_2_S-specific probe P3, gp120 (500 pM) (*p* < 0.001), morphine (1 μM) (*p* < 0.0001), gp120 plus morphine (*p* < 0.0001), and FAC (50 μM) significantly decreased levels of H_2_S in endolysosomes (Figure 6(A–D)); no significant additive effects were observed when gp120 was added together with morphine (Figure 6(A,B)). DFO (50 μM) significantly (*p* < 0.0001) blocked gp120-, morphine-, gp120 plus morphine-, and FAC-induced decreases in levels of H_2_S. Cells cultured in FBS-free media (FBS 0%) significantly (*p* < 0.0001) increased endolysosome H_2_S (Supplementary Figure S6A, B). Hi-gp120 (500 pM) did not significantly affect levels of endolysosome H_2_S (Supplementary Figure S6A, B).

**Figure 7.**
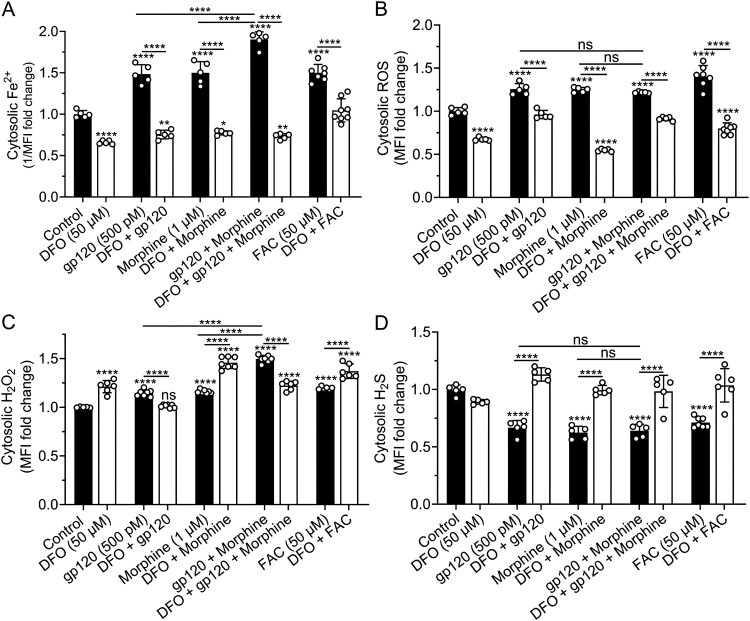
Effects of DFO on gp120-, morphine-, and iron supplementation-induced changes in levels of cytosolic Fe^2+^, ROS, H_2_O_2_, and H_2_S. (A–D) Measurement of cytosolic Fe^2+^, ROS, H_2_O_2_ and H_2_S levels in SH-SY5Y cells pretreated with DFO, followed by treatment with gp120, morphine, gp120 plus morphine, or FAC. (A) Cytosolic Fe^2+^ levels were measured using the quenching dye PhenGreen FL diacetate (PGFLDA) and data were represented as the reciprocal of mean fluorescence intensity (1/MFI). (B–C) Cytosolic ROS, H_2_O_2_, and H_2_S levels were measured using CM-H2DCFDA, H_2_O_2_ assay kit, SF7-AM, respectively, and data Represented as fold changes in MFI relative to control. Data were shown as mean ± SD, with individual data points (*n* = 5–6) were included on each bar. One-way ANOVA with Tukey’s multiple comparison test was used for statistical analyses. ns = non-significant, *p < 0.05, **p < 0.01, ****p < 0.0001.

Effects of gp120, morphine, gp120 plus morphine, and iron supplementation on cytosolic levels of Fe^2+^, ROS, H_2_O_2,_ and H_2_S. gp120 (500 pM), morphine (1 μM), gp120 plus morphine, as well as FAC (50 μM) significantly (*p* < 0.0001) increased levels of cytosolic Fe^2+^, ROS, and H_2_O_2_, and significantly (*p* < 0.0001) decreased levels of cytosolic H_2_S (Figure 7(A–D)). gp120 plus morphine, in comparison to gp120 or morphine alone, produced significantly (*p* < 0.0001) higher cytosolic levels of Fe^2+^ (Figure 7(A)) and H_2_O_2_ (Figure 7(C)), but not levels of ROS (Figure 7(B)) and H_2_S (Figure 7(D)). Preincubation of SH-SY5Y cells for 1 h with DFO (50 μM) significantly (*p* < 0.0001) decreased cytosolic levels of Fe^2+^ (Figure 7(A)) and ROS (Figure 7(B))_,_ and significantly (*p* < 0.0001) blocked gp120-, morphine-, and FAC-induced changes in Fe^2+^, ROS, and H_2_S (Figure 7(A,B,D)). DFO (50 μM) significantly (*p* < 0.0001) increased cytosolic levels of H_2_O_2_, significantly (*p* < 0.0001) increased morphine-induced increases in levels of H_2_O_2_, significantly (*p* < 0.0001) decreased gp120 plus morphine-induced increases in H_2_O_2_, and significantly (*p* < 0.0001) increased FAC-induced increases in H_2_O_2_ (Figure 7(C)). Similar results for cytosolic Fe^2+^ and ROS levels were also observed in U87MG cells (data not shown). Hi-gp120 (500 pM) did not significantly affect levels of cytosolic Fe^2+^, ROS, H_2_O_2,_ and H_2_S (Supplementary Figure S7A-D). Extracellular levels of H_2_S were not significantly affected by gp120 and morphine alone or in combination (Supplementary Figure S7E).

**Figure 8.**
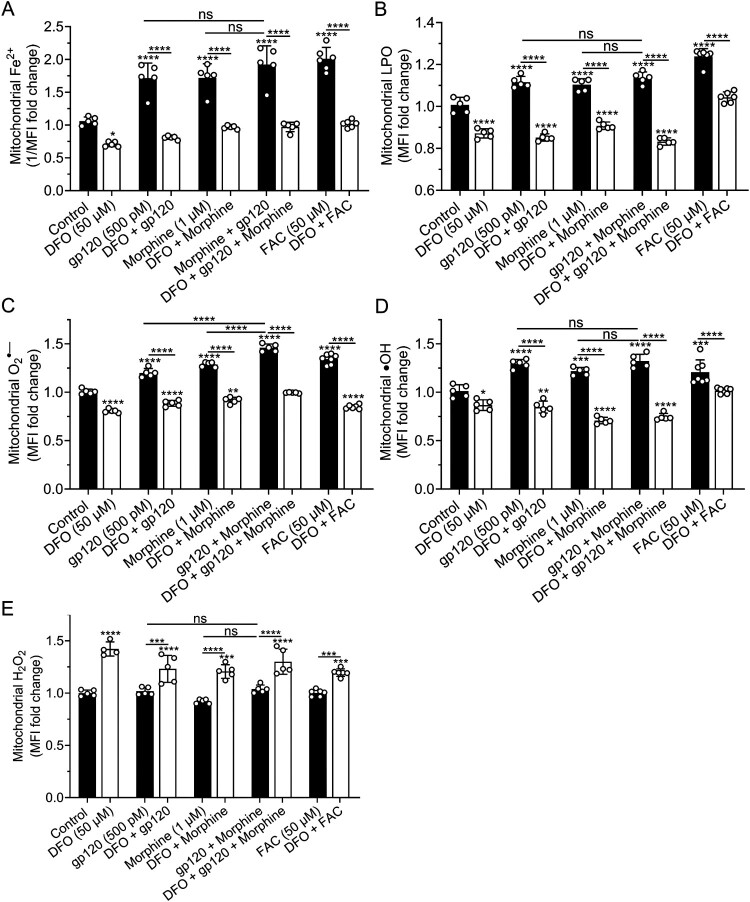
Effects of DFO, gp120, morphine, and iron supplementation on levels of mitochondrial Fe^2+^, LPO, O_2_^•−^, ^•^OH, and H_2_O_2_. (A–E) Measurement of mitochondrial Fe^2+^, LPO, O_2_^•−^, ^•^OH, and H_2_O_2_ levels in SH-SY5Y cells pretreated with DFO, and then treatment with gp120, morphine, gp120 plus morphine, or FAC. (A) Levels of mitochondrial Fe^2+^ were measured with the quenching dye RDA; data were represented as the reciprocal of mean fluorescence intensity (1/MFI). (B-E) Levels of mitochondrial LPO, O_2_^•−^, ^•^OH and H_2_O_2_ were measured using MitoPerOx, MitoSOX, OH580 ^•^OH kit, and MitoPY1, respectively; data were presented as fold changes in MFI relative to control. Data were shown mean ± SD, with individual data points (*n* = 5–7) included on each bar. One-way ANOVA with Tukey’s multiple comparison test was used for statistical analyses. ns = non-significant, *p < 0.05, **p < 0.01, ***p < 0.001, ****p < 0.0001.

Effects of DFO, gp120, morphine, and iron supplementation on levels of mitochondrial Fe^2+^, LPO, O_2_^•−^, ^•^OH, and H_2_O_2_. gp120 (500 pM), morphine (1 μM), gp120 plus morphine, as well as FAC (50 μM) significantly (*p* < 0.0001) increased levels of mitochondrial Fe^2+^, LPO, O_2_^•−^, and ^•^OH (Figure 8(A–D)). gp120 plus morphine, in comparison to gp120 or morphine alone, produced significantly (*p* < 0.0001) higher levels of O_2_^•−^ (Figure 8(C)), but not Fe^2+^ (Figure 8(A)), LPO (Figure 8(B)), and ^•^OH (Figure 8(D)). DFO (50 μM) significantly decreased mitochondrial levels of Fe^2+^ (Figure 8(A), *p* < 0.02), LPO (Figure 8(B), *p* < 0.0001), O_2_^•−^ (Figure 8(C), *p* < 0.0001) and ^•^OH (Figure 8(D), *p* < 0.023), and significantly (*p* < 0.0001) blocked gp120-, morphine-, gp120 plus morphine-, and FAC-induced increased levels of Fe^2+^, LPO, O_2_^•−^, and ^•^OH (Figure 8(A–D)). These findings for mitochondrial Fe^2+^ and O_2_^•−^ levels were also observed in U87MG cells (data not shown). gp120, morphine, gp120 plus morphine, and FAC did not significantly affect levels of mitochondrial H_2_O_2_ but DFO alone or in combination with these treatments significantly increased levels of mitochondrial H_2_O_2_ (Figure 8(E)). Hi-gp120 (500 pM) did not significantly affect mitochondrial levels of Fe^2+^, LPO, O_2_^•−^, ^•^OH, and H_2_O_2_ (Supplementary Figure S8A-E).

**Figure 9.**
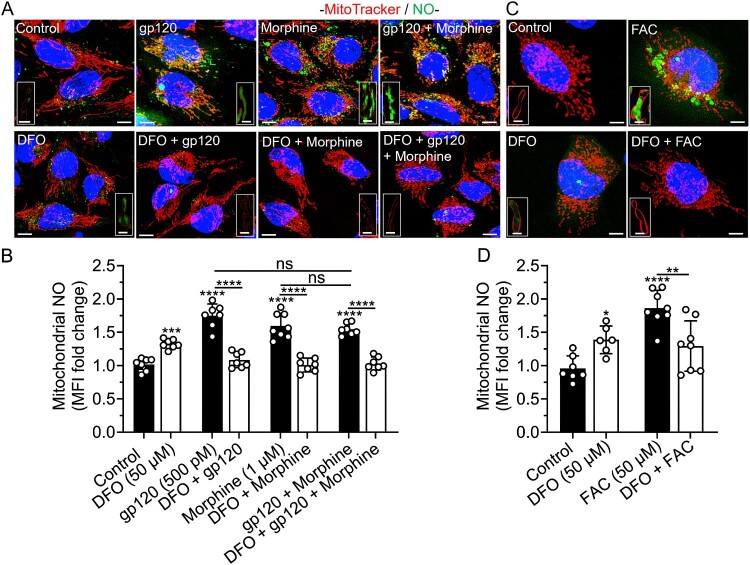
DFO blocked gp120-, morphine-, iron supplementation- induced increases in levels of mitochondrial NO. (A, C) Representative confocal images of SH-SY5Y cells pretreated for 1 h with DFO (50 μM) or water (control) and then treated for 1 h with gp120 (500 pM), morphine (1 μM), gp120 plus morphine, and FAC (50 μM) prior to staining for NO with DAF-FM DA (green), mitochondria with MitoTracker Red (red), and nuclei with Hoechst 33342 (blue). Scale bars = 10 μm. Boxed inserts are magnified mitochondria containing NO. Scale bar = 1 μm. (B, D) Semi quantitative changes in mitochondrial NO were determined using Imaris 3D software and data were represented as fold changes of mean fluorescence intensity (MFI) inside MitoTracker-positive mitochondria. Data were presented as mean ± SD, with individual data points (*n* = 6–8) included on each bar. Each data point (n) represents MFI of DAF-FM DA staining inside mitochondria from multiple cells. A minimum of 120 cells per condition from two biological replicates were analyzed. One-way ANOVA with Tukey’s multiple comparison test was used for statistical analyses. *p < 0.05, **p < 0.01, ***p < 0.001, ****p < 0.0001.

**Figure 10.**
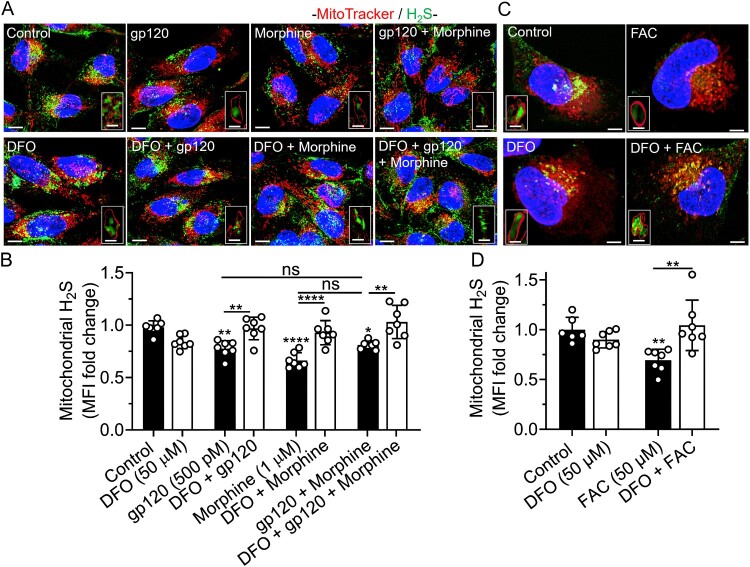
DFO blocked gp120-, morphine-, and iron supplementation-induced decreases in mitochondrial levels of H_2_S. (A, C) Representative confocal images of SH-SY5Y cells pretreated for 1 h with DFO (50 μM) or water (control) and then treated for 1 h with gp120 (500 pM), morphine (1 μM), gp120 plus morphine, and FAC (50 μM) prior to staining for H_2_S with P3 probe (green), for mitochondria with MitoTracker Red (red), and for nuclei with Hoechst 33342 (blue). Scale bars = 10 μm. Boxed inserts are magnified mitochondria containing H_2_S. Scale bars = 1 μm. (B, D) Semi-quantitative changes in mitochondrial H_2_S were determined using Imaris 3D software and data were represented as fold changes in mean fluorescence intensity (MFI) inside MitoTracker-positive mitochondria. Data were shown as (mean ± SD) and individual data points (*n* = 6–7) were illustrated. Each data point (n) represents MFI of P3 staining inside mitochondria from multiple cells. A minimum of 61 cells per condition from two biological replicates were analyzed. One-way ANOVA with Tukey’s multiple comparison test was used for statistical analyses. ns = non-significant, *p < 0.05, **p < 0.01, ****p < 0.0001.

DFO blocked gp120-, morphine-, gp120 plus morphine-, and iron supplementation-induced increases in mitochondrial NO and decreases in mitochondrial H_2_S levels. gp120 (500 pM), morphine (1 μM), gp120 plus morphine, and FAC (50 μM) significantly (*p* < 0.0001) increased levels of mitochondrial NO (Figure 9(A–D)) and significantly decreased levels of mitochondrial H_2_S (Figure 10(A–D)). DFO (50 μM) alone significantly (*p* < 0.001) increased levels of mitochondrial NO and significantly (*p* < 0.0001) blocked gp120-, morphine-, gp120 plus morphine-, and FAC-induced changes in levels of mitochondrial NO and H_2_S (Figures 9 and 10(A–D)). gp120 plus morphine had no significant additive effects on NO and H_2_S levels (Figures 9 and 10(A,B)). Hi-gp120 (500 pM) did not significantly affect levels of mitochondrial NO and H_2_S (Supplementary Figure S9 and S10A,B). FBS-free media (FBS 0%) used as positive control, significantly (*p* < 0.0001) increased mitochondrial H_2_S levels (Supplementary Figure S10A, B).

**Figure 11.**
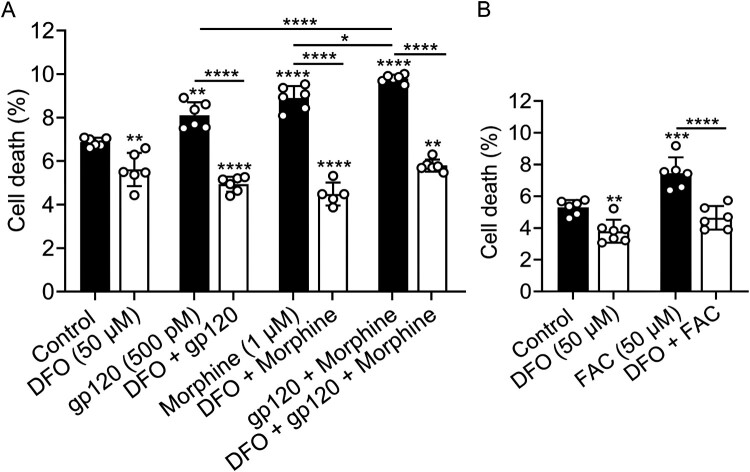
DFO blocked gp120-, morphine- and iron supplementation-induced cell death. (A, B) SH-SY5Y cells were pretreated for 1 h with DFO (50 μM) prior to being treated for 24 h with water (control), gp120 (500 pM), morphine (1 μM), gp120 plus morphine, or FAC (50 μM) and staining with propidium iodide (PI). Data were collected using flow cytometry and presented as percent cell death. Data were represented as mean ± SD with individual data points (*n* = 5–6) included on each bar. One-way ANOVA with Tukey’s multiple comparison test was used for statistical analyses. *p < 0.05, **p < 0.01, ***p < 0.001, ****p < 0.0001.

DFO blocked gp120-, morphine-, and iron supplementation-induced cell death: gp120 (500 pM), morphine (1 μM), gp120 plus morphine, and FAC (50 μM) significantly increased cell death (Figure 11(A,B)); significant additive increases were observed when gp120 was added in combination with morphine. DFO (50 μM) significantly (*p* < 0.01) decreased cell death and significantly (*p* < 0.0001) blocked gp120-, morphine-, gp120 plus morphine-, and FAC-induced cell death (Figure 11(A,B)). Similar results were found using U87MG cells (data not shown). Hi-gp120 (500 pM) did not significantly affect levels of cell death (Supplementary Figure S11).

## Discussion

**Figure 12.**
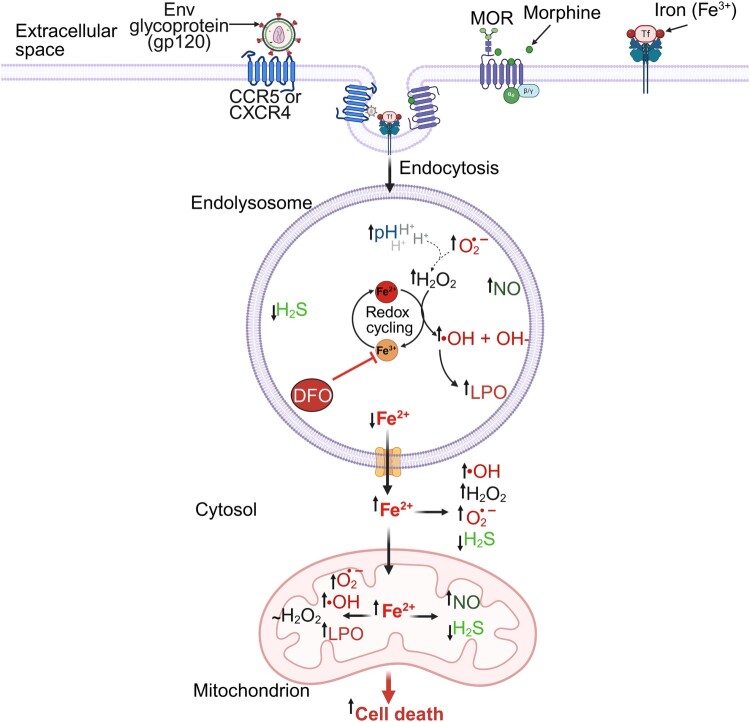
Summary illustration. gp120-, morphine-, and iron are internalized via endocytosis and enter endolysosomes. These agents induce endolysosome de-acidification and disrupt endolysosome iron (Fe^2+^) and reactive species interactome (RSI) homeostasis. This dysfunction leads to the release of endolysosome Fe^2+^ into the cytosol, resulting in increased Fe^2+^ levels and RSI disruption in both the cytosol and mitochondria, ultimately inducing cell death. The endolysosome-specific iron chelator DFO blocked these effects, highlighting the central role of endolysosome iron in RSI disruption and gp120-, morphine-, and iron-induced neurotoxicity.

Implicated in the pathogenesis of neurodegenerative disorders, including HAND, are iron overload-induced oxidative damage and elevated levels of reactive species [5,13,30,31]. We demonstrated previously that both gp120 and morphine disrupt iron metabolism and increase ROS levels [7,32]. Because iron levels are elevated in patients with HAND and iron influences redox homeostasis [33–35], we extended our studies by determining the extent to which gp120 and morphine alone and in combination as well as iron supplementation induces neural cell death, and affects levels of Fe^2+^ and the RSI in endolysosomes, cytosol, and mitochondria. Here, we demonstrated that gp120, morphine, gp120 plus morphine, and iron supplementation de-acidified endolysosomes, decreased endolysosome Fe^2+^ levels, increased levels of endolysosome ROS, LPO and NO, decreased levels of endolysosome H_2_S, increased levels of cytosolic Fe^2+^ and ROS, decreased cytosolic H_2_S levels, increased levels of mitochondrial Fe^2+^, ROS, LPO and NO, decreased mitochondrial H_2_S levels, and induced cell death; effects blocked by the iron chelator DFO (Figure 12).

Iron is an essential element involved in the control of many physiological processes [35]. Endolysosomes function as signaling hubs, have readily releasable stores of Fe^2+^, and are ‘master regulators of cellular iron homeostasis’ [15,16]. Iron stores in endolysosomes are sufficiently large to account for lysosome stress response (LSR)-induced increases in levels of cytosolic and mitochondrial Fe^2+^ as well as cell death [7,32,36,37].

Endolysosome de-acidification is central to LSR and controlling iron levels and redox homeostasis [15,20,32]. gp120 and other viral proteins, opioids, and clinically used weak-base drugs de-acidify endolysosomes, decrease endolysosome Fe^2+^ levels, and increase levels of cytosolic and mitochondrial Fe^2+^ and ROS [7,32,36,38]. Here, we observed that gp120 and morphine alone and in combination as well as iron supplementation de-acidified endolysosomes and decreased levels of endolysosome Fe^2+^; findings consistent with previous reports [32]. Although we did not specifically examine mechanisms, it is likely that TRPML1 channels are involved because they become more active as endolysosomes are de-acidified [39]. gp120 is an arginine-rich molecule [40] that enters endolysosomes via endocytosis and therein may contribute to de-acidification by buffering luminal protons as observed here. In addition, gp120 induces oxidative stress and activates TRPML1 channels – both of which disrupt endolysosome pH [41–43]. Opioid agonists such as morphine act through G-protein-coupled receptors to reduce intracellular cAMP levels [44,45]. cAMP is a known regulator of endolysosome pH by affecting v-ATPase [46], a proton pump responsible for endolysosome acidification. Thus, the effects of gp120, morphine and FAC on endolysosome pH likely stem from multiple converging mechanism including proton buffering, oxidative stress, and impaired v-ATPase function. Endolysosome de-acidification induced by these insults likely leads to TRPML1 activation, decreases in endolysosome Fe^2+^ levels, and cell death.

Decreased levels of endolysosome Fe^2+^ have been linked to increased levels of ROS, and RNS, and increased endolysosome membrane lipid peroxidation [47]. Here, we found that gp120 and morphine alone and in combination as well as FAC significantly increased endolysosome levels of ROS, LPO, and NO. Mechanisms underlying these effects likely include the induction of oxidative stress through activation of NADPH oxidases (NOX) because NOX2 activation increases the transport of electrons from cytosolic NADPH into the endolysosome lumen, reduces O_2_ to O_2_^•−^, increases H_2_O_2_ and hydroxyl radicals through Fenton-like reactions, causes endolysosome de-acidification, and increases endolysosome LPO levels [48,49]. Indeed, gp120 and morphine both increase membrane lipid peroxidation and cause oxidative damage in brain [11,50,51]. Moreover, in endolysosomes, activation of inducible nitric oxide synthase (iNOS) increased levels of NO and de-acidified endolysosomes [52]. Fe^2+^, NO, and O_2_^•−^ can induce peroxynitrite-mediated-lysosomal lipid peroxidation [53,54] and endolysosome de-acidification as we observed here.

Reactive sulfur species (RSS) particularly H_2_S can improve mitochondrial respiration, reduce mitochondrial ROS production, promote mitochondrial ATP production, attenuate ferroptosis, exert anti-inflammatory effects [55,56], and help maintain balanced levels of mitochondrial ROS, LPO, and RNS [57]. On the other hand, gp120, morphine, and FAC can increase mitochondrial iron, decrease mitochondrial ATP, and can promote oxidative stress, inflammation, and apoptotic cell death [5,7,32, p.120]. Of relevance to the pathogenesis of HAND, depletion of endogenous H_2_S can promote HIV-1 reactivation [58] and here we found that DFO blocked gp120-, morphine-, and FAC-induced decreases in mitochondrial H_2_S levels. Increased levels of cytosolic Fe^2+^ can cause ROS production via Fenton-like reactions and increased ROS can oxidize H_2_S to RSS thereby decreasing cytosolic H_2_S [59,p.1]. In addition, gp120, morphine, and FAC decreased endolysosome levels of H_2_S and this reduction caused lipid peroxidation and ferroptosis [20,60]. Because endolysosomes are enriched in cystine [61] and entry of cystine into endolysosomes is pH-dependent, decreases in H_2_S are likely due to endolysosome de-acidification induced decreases in endolysosome cystine levels [20,62].

Endolysosome acidification is essential for maintaining iron and redox homeostasis – and vice versa – as iron dysregulation can also disrupt luminal pH [62,63]. Consistent with our findings, endolysosome de-acidification led to decreased endolysosome Fe^2+^ levels. Treatment with the endolysosome-specific iron chelator DFO resulted in endolysosome acidification and decreased endolysosome Fe^2+^. Within endolysosomes, iron exists in a redox equilibrium between Fe^2+^ and Fe^3+^ that is maintained in part by STEAP3 – a pH-dependent ferrireductase that reduces Fe^3+^ to Fe^2+^ under acidic conditions [15,63]. By chelating Fe^3+^, DFO disrupt this equilibrium thereby decreasing Fe^2+^ levels [64,65]. Additionally, DFO-induced acidification may further stabilize Fe^3+^ and inhibit its reduction. Together, these effects disrupt the Fe^3+^/Fe^2+^ balance, limit levels of redox-active Fe^2+^, and protect endolysosomes from iron-mediated oxidative damage.

A direct link between endolysosome pH and Fe^2+^ and their effects on RSI is further supported by our findings that the endolysosome-specific iron chelator DFO effectively blocked endolysosome de-acidification and the effects of gp120, morphine, and FAC on RSI. Previously, it was reported that ROS [66], the LPO end product 4-HNE [67], and NO all de-acidified endolysosomes by directly inhibiting v-ATPase activity [47,52] thus linking together endolysosome Fe^2+^ dyshomeostasis with effects on the RSI and disruption of normal transmembrane pH gradients [68].

Treatment with gp120 and morphine alone and in combination as well as FAC increased cytosolic H_2_O_2_ levels; previously gp120 increased intracellular H_2_O_2_ levels [69]. DFO alone increased cytosolic H_2_O_2_ levels, but it blocked gp120-induced H_2_O_2_ increases but not those with morphine and FAC. Combining DFO with either morphine or FAC further elevated cytosolic H_2_O_2_ levels compared to either treatment alone. These findings suggest that gp120-induced H_2_O_2_ production is largely iron-dependent and sensitive to iron chelation, whereas morphine and FAC may influence H_2_O_2_ production though multiple converging mechanisms, both iron-dependent and iron-independent.

Previous studies showed that iron supplementation with FeSO_4_ did not affect mitochondrial H_2_O_2_ levels [70]. H_2_O_2_ promoted iron uptake, increased cytosolic and mitochondrial Fe^2+^ levels, and regulated iron-response proteins [71,72]. Here, gp120, morphine, and FAC did not increase mitochondrial H_2_O_2_ levels, but DFO alone and in combination with these treatments, increased mitochondrial H_2_O_2_ levels. This may reflect a compensatory redox signaling response to iron depletion, as DFO reduced mitochondrial Fe^2+^ below basline. Such depletion could trigger oxidative stress, leading to H_2_O_2_ production and activation of protective antioxidant pathways such as SKN-1/Nrf2 to maintain redox and iron homeostasis [73,74]. Although the mechanisms for this are unclear at present, we propose that DFO-induced H_2_O_2_ serves as an adaptive redox signaling trigged by iron chelation. DFO has been reported to activate Nrf2-dependent antioxidant responses [74] and H_2_O_2_ is a known activator of Nrf2 pathway [75]. Thus, cells may increase H_2_O_2_ production as a response to iron chelation as part of compensatory mechanisms to restore cellular redox and iron homeostasis. Future studies will be needed to test this hypothesis and define pathways linking iron chelation to H_2_O_2_ signaling to Nrf2-antioxidant responses. Consistent with this protective effect, DFO also blocked gp120-, morphine-, gp120 plus morphine-, and FAC-induced increases in mitochondrial Fe^2+^, ROS (^•^OH, O_2_^•^**^−)^**, LPO, and NO as well as a decrease in H_2_S. Previously, it was reported that DFO blocked mitochondrial Fe^2+^ accumulation and increases in ROS and LPO [32,76].

NO plays important roles in regulating iron, mitochondrial function, and has been implicated in the pathogenesis of neurodegenerative disorders including HAND [6,77,p.1]. NO controls cellular iron metabolism post-translationally; increased IRP1 RNA binding activity leads to increases in TfR mRNA levels and decreases in cytosolic ferritin synthesis [12,78]. Iron supplementation in neurons increased levels of mitochondrial peroxynitrite, protein tyrosine nitration, and membrane lipid peroxidation [79]. Elsewhere and here we showed that DFO blocked gp120-, morphine-, gp120 plus morphine-, and FAC-induced increases in mitochondrial Fe^2+^, NO, and depolarization of mitochondrial membrane potential (ΔΨm) [7,32,80,81]. DFO can polarize ΔΨm [32], increase mitochondrial NO, and decrease mitochondrial Fe^2+^ below basal levels.

gp120, morphine, and iron overload can induce oxidative stress-mediated cell death [43,81–83] and endolysosome iron dyshomeostasis has been linked to gp120- and morphine-induced cell death [19,32] by non-apoptotic and ferroptotic mechanisms [84]. Here, we confirmed and extended these findings by demonstrating the involvement of RSI in gp120-, morphine-, and FAC-induced increases in neural cell death. The modest increase in cell death we observed is consistent with previous reports of gp120- and morphine-induced neurotoxicity, which vary depending on concentration, exposure duration, neuronal subtypes, and the method used to assess cell death [85–88]. Notably, similar to previous findings that gp120 and morphine act additively or synergistically to induce neuronal death [89], we found that gp120- and morphine-induced increases in cell death were additive when co-applied. The modest but consistent increase in cell death was effectively blocked by DFO, indicating that endolysosome iron dysregulation contributes to the observed cell death.

While our study shows that gp120, morphine and iron supplementation contribute to disruptions in endolysosome, cytosolic and mitochondrial Fe^2+^ and RSI homeostasis, we did not specifically investigate the chronological order in which these events occur. Based on our findings and previous studies, endolysosome Fe^2+^ dyshomeostasis may act as a central amplifier of RSI disruption and serve as an early upstream event. This dysfunction likely precedes and contributes to cytosolic and mitochondrial Fe^2+^ and RSI imbalance, ultimately leading to cell death. This is further supported by our observation that the endolysosome-specific iron chelator DFO effectively blocked these effects. Future studies using time-resolved and compartment-specific approaches will be needed to further define the order of these events.

Overall, our findings suggest that endolysosome iron plays a central role in mediating cellular response to external insults-such as gp120, morphine and excess iron-by regulating RSI homeostasis and contributing to cellular injury. Therefore, targeting or fine-tuning this Fe^2+^ pool may provide therapeutic benefit against HAND and other neurodegenerative disorders. Indeed, DFO is being promoted as a therapeutic against neurodegenerative diseases [90,91].

## Supplementary Material

Supplementary Material.pdf

## Data Availability

In addition to the data included in the manuscript and the supplementary data sections, additional information is available from the corresponding author upon reasonable request.
