# Supplementary material for: Involvement of endolysosome iron in HIV-1 gp120-, morphine-, and iron supplementation-induced disruption of the reactive species interactome and induction of neurotoxicity
Source: Redox Rep. 2025 Aug 21;30(1):2546496. doi: 10.1080/13510002.2025.2546496 (PMC12372519; doi:10.1080/13510002.2025.2546496)
Supplement: Supplementary Material.pdf [file YRER_A_2546496_SM9349.pdf]

Supplementary Materials

Involvement of endolysosome iron in HIV-1 gp120-, morphine-, and iron supplementation-induced disruption of the reactive species interactome and induction of neurotoxicity

Nirmal Kumar, Peter W. Halcrow, Darius N.K. Quansah, Braelyn Liang, Olimpia Meucci and Jonathan D. Geiger*

***Corresponding author:**

Jonathan D. Geiger, Ph.D., Chester Fritz Distinguished Professor, Department of Biomedical Sciences, University of North Dakota School of Medicine and Health Sciences, 504 Hamline Street Room 110, Grand Forks, North Dakota 58203, (701) 777-2183; jonathan.geiger@und.edu

The PDF file includes:

Supplementary Figure S1 to S12

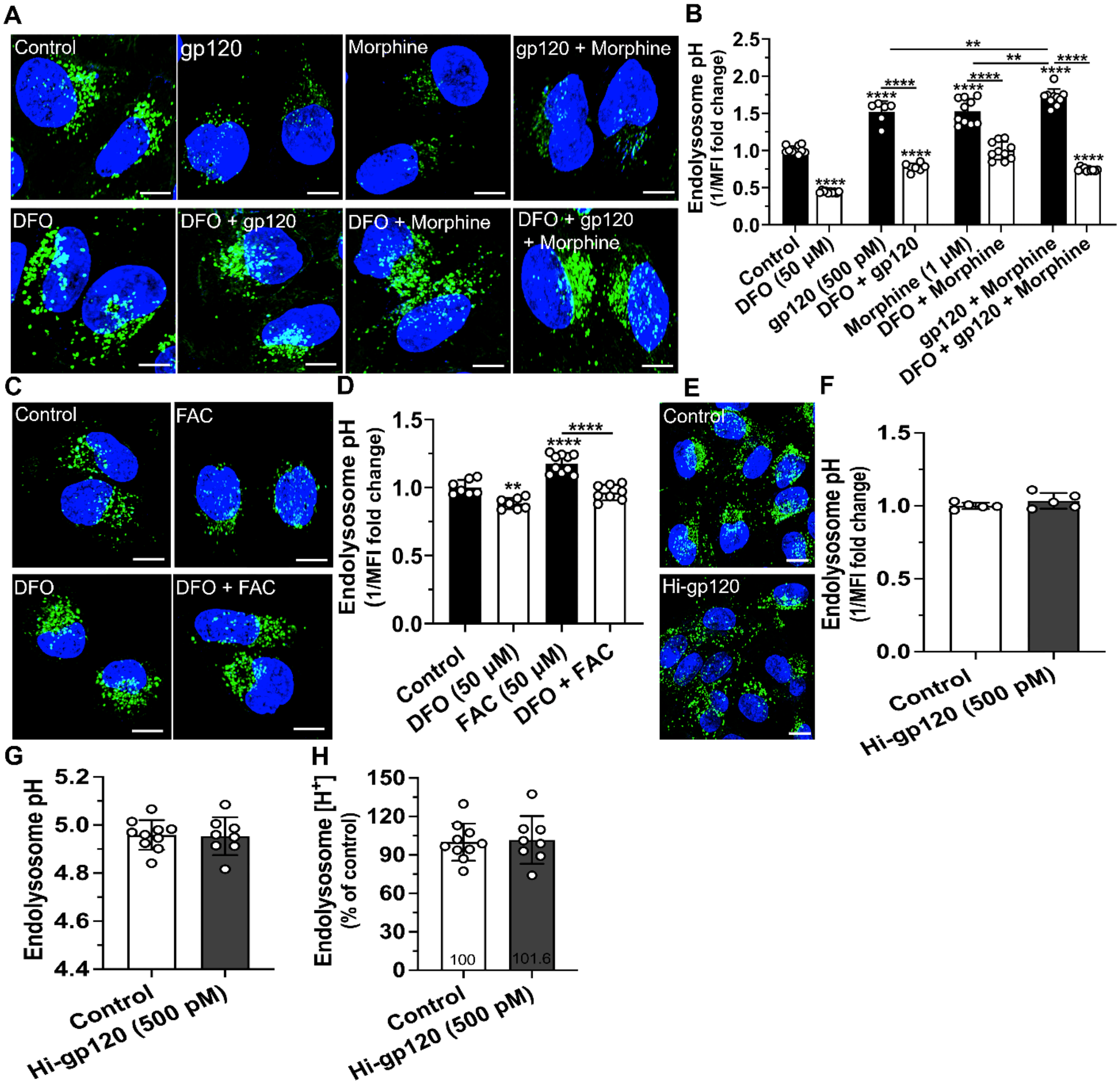
A
Control
gp120
Morphine
gp120 + Morphine
DFO
DFO + gp120
DFO + Morphine
DFO + gp120 + Morphine
B
Endolysosome pH (1/MFI fold change)
Control
DFO (50 μM)
gp120 (500 pM)
DFO + gp120
Morphine (1 μM)
DFO + Morphine
gp120 + Morphine
DFO + gp120 + Morphine
C
Control
FAC
DFO
DFO + FAC
D
Endolysosome pH (1/MFI fold change)
Control
DFO (50 μM)
FAC (50 μM)
DFO + FAC
E
Control
Hi-gp120
F
Endolysosome pH (1/MFI fold change)
Control
Hi-gp120 (500 pM)
G
Endolysosome pH
Control
Hi-gp120 (500 pM)
H
Endolysosome [H+] (% of control)
100
101.6
Control
Hi-gp120 (500 pM)

Supplementary Figure S1. Effect of gp120, morphine, FAC, DFO and heat-inactivated gp120 on endolysosome pH. (A, C, E) Representative images of SH-SY5Y cells pretreated for 1 h with DFO (50 μM) or water (control) and then treated for 1 h with gp120 (500 pM), heat-inactivated gp120 (Hi-gp120), morphine (1 μM), gp120 plus morphine, and FAC (50 μM). Following treatments, cells were stained with LysoSensor Green DND-189 (green) for endolysosome pH and the nuclear stain Hoechst 33342 (blue). Scale bars = 10 μm. **(B, D, F)** Semi-quantitative changes in pH were determined from images using Imaris 3D software, and data were transformed and represented as the reciprocal of mean fluorescence intensity fold change (1/MFI). **(G)** Quantitative measurement of endolysosome pH with the ratiometric dye LysoSensor Yellow/Blue DND-160 and data were shown as pH values. **(H)** H^+ concentrations were not significantly affected by Hi-gp120. Data were shown as mean ± SD, with individual data points (n = 5-10) included on each bar. In Panels B, D and F, each data point (n) represents MFI from multiple cells within a field of view. In panels G and H, each data point (n = 8-10) represents the mean pH measurement of endolysosomes from cells in a single well of a 96-well plate. Two-tailed Student's *t*-test or one-way ANOVA with Tukey's multiple comparison test was used for statistical analyses. $^{**}p<0.01$, $^{****}p<0.0001$

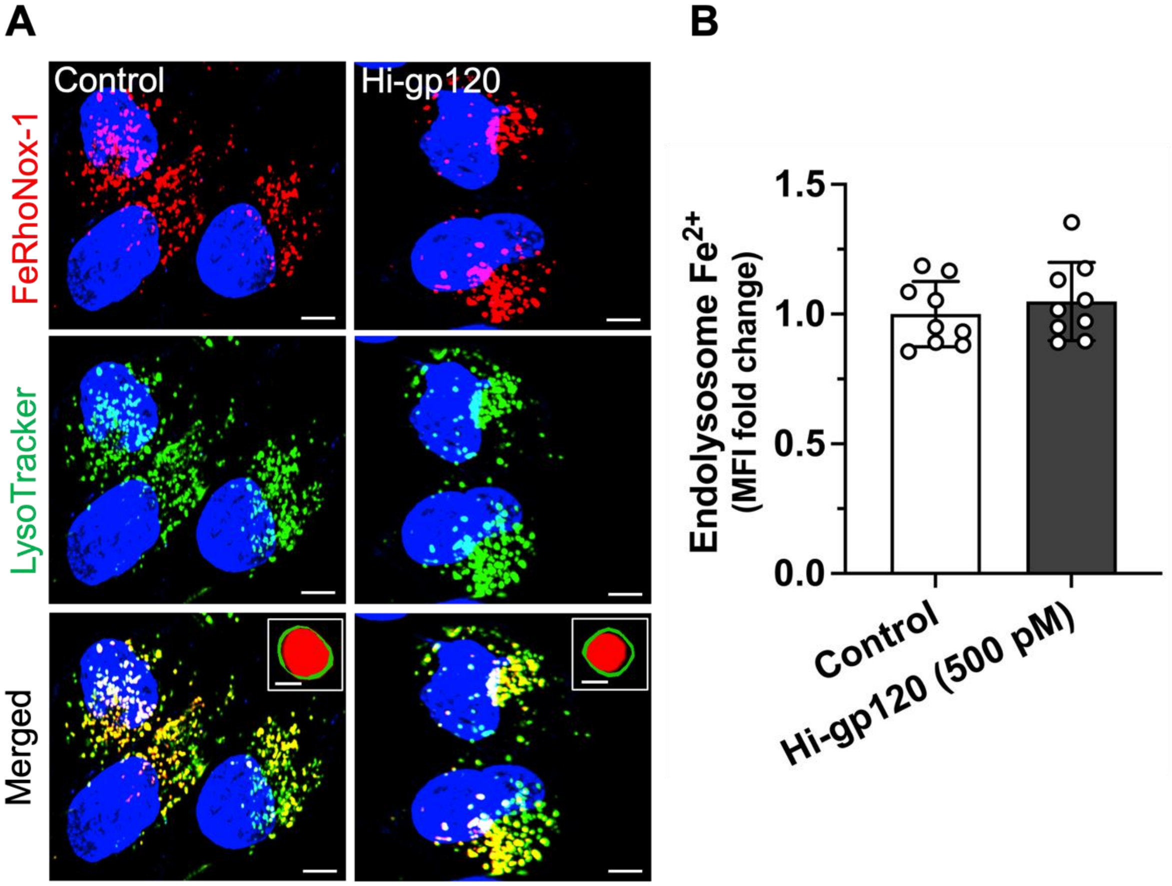
A
Control
Hi-gp120
FeRhoNox-1
LysoTracker
Merged
B
Endolysosome Fe^{2+}
(MFI fold change)
1.5
1.0
0.5
0.0
Control
Hi-gp120 (500 pM)

Supplementary Figure S2. Heat-inactivated gp120 did not significantly affect levels of endolysosome Fe^{2+}. (A) Representative images of SH-SY5Y cells treated for 1 h with water (control) or Hi-gp120 (500 pM) and stained for Fe^{2+} (FeRhonox-1, red), endolysosomes (LysoTracker Green, green), and nuclei (Hoechst 33342, blue). Scale bars =10 μm. Boxed inserts are magnification of single composite endolysosomes containing Fe^{2+}. Scale bar = 1 μm. **(B)** Images were quantified using Imaris 3D software and data were represented as fold-changes of mean fluorescence intensity (MFI) of FeRhonox-1 staining inside of LysoTracker-positive endolysosomes. Data were shown as mean ± SD, with individual data points (n = 9) included on each bar. Each data point (n) represents MFI of FeRhonox-1 staining inside LysoTracker-positive endolysosomes from multiple cells. A minimum of 42 cells per condition were analyzed using Imaris 3D software. Two-tailed Student's *t*-test was used to analyze the data.

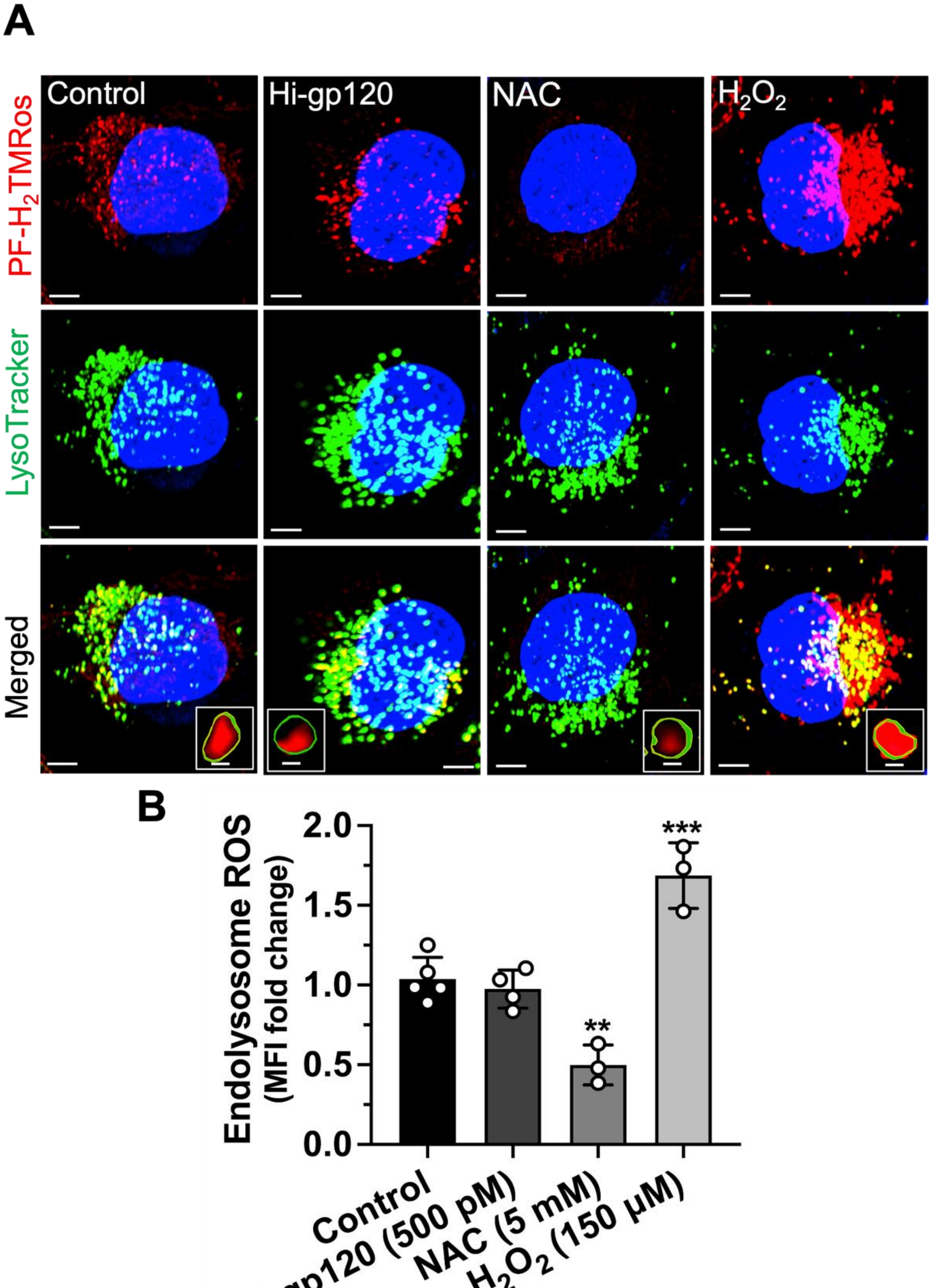
A
Control
Hi-gp120
NAC
H_2O_2
PF-H_2TMRos
LysoTracker
Merged
B
Endolysosome ROS
(MFI fold change)
2.0
1.5
1.0
0.5
0.0
***
**
Control
Hi-gp120 (500 pM)
NAC (5 mM)
H_2O_2 (150 μM)

Supplementary Figure S3. Effects of Hi-gp120, NAC, and H_2O_2 on levels of endolysosome ROS. (A) Representative images of SH-SY5Y cells treated with water (control), Hi-gp120 (500 pM), NAC (5 mM), and H_2O_2 (150 μM) for 1 h and stained for ROS with PF-H_2TMRos (red), endolysosomes (LysoTracker Green, green), and nuclei (Hoechst 33342, blue). Scale bars = 10 μm. Boxed inserts are magnifications of single composite endolysosomes containing ROS. Scale bar = 1 μm. **(B)** Images were quantified using Imaris 3D software and data were represented as fold-changes of mean fluorescence intensity (MFI) of PF-H_2TMRos in LysoTracker-positive endolysosomes. Data were presented as mean ± SD, with individual data points (n = 3-5) shown on each bar. Each data point (n) represents MFI of PF-H_2TMRos staining inside LysoTracker-positive endolysosomes from multiple cells within a field of view. A minimum of 54 cells per condition were analyzed using Imaris 3D software. One-way ANOVA with Tukey's multiple comparison test was used for statistical analyses. $^{**}p < 0.01$, $^{***}p < 0.001$

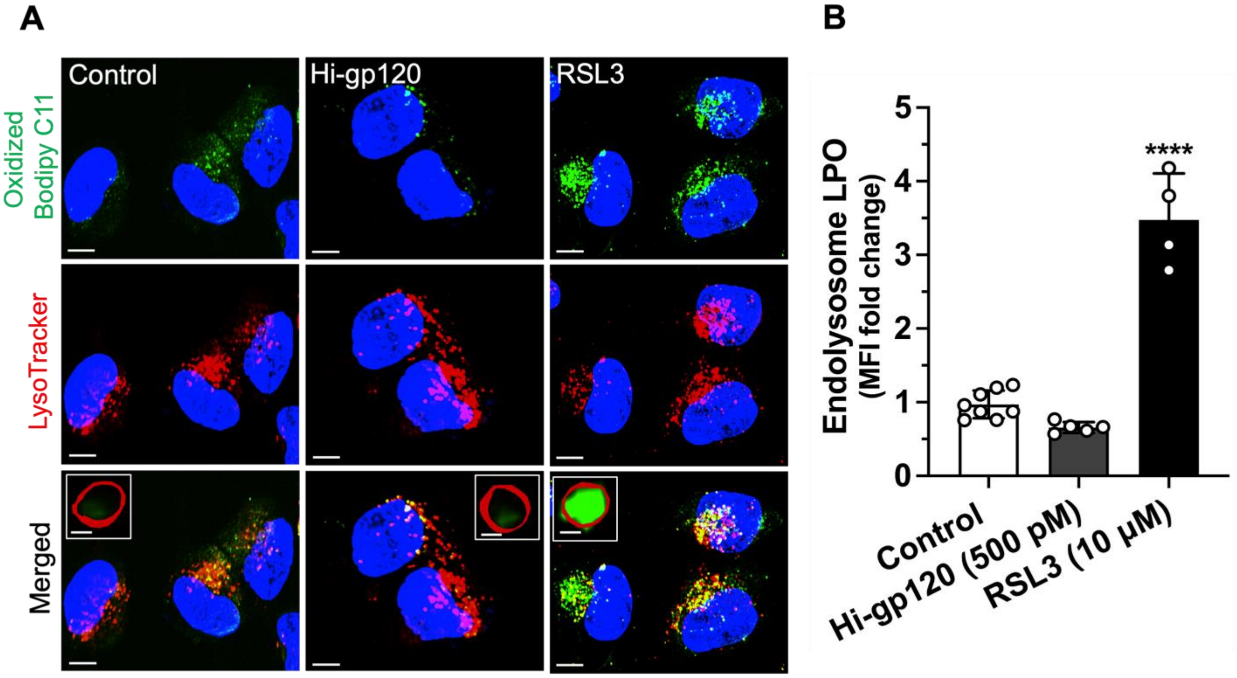
A
B
Control
Hi-gp120
RSL3
Oxidized Bodipy C11
LysoTracker
Merged
Endolysosome LPO
(MFI fold change)
5
4
3
2
1
0
****
Control
Hi-gp120 (500 pM)
RSL3 (10 μM)

Supplementary Figure S4. Heat-inactivated gp120 did not significantly affect levels of endolysosome lipid peroxidation (LPO). **(A)** Representative images of SH-SY5Y cells treated with water (control), Hi-gp120 (500 pM) and RSL3 (10 μM) for 1 h and stained for LPO with Bodipy 581/591 C11 (green), endolysosomes (LysoTracker far red, red), and nuclei (Hoechst 33342, blue). Scale bars = 10 μm. Boxed inserts are magnifications of single composite endolysosomes containing Bodipy C11. Scale bar = 1 μm. **(B)** Images were quantified using Imaris 3D software and data were represented as fold-changes of mean fluorescence intensity (MFI) of oxidized Bodipy C11 in LysoTracker-positive endolysosomes. Data were represented as mean ± SD, with individual data points (n = 4-8) shown on each bar. Each data point (n) represents MFI of oxidized Bodipy C11 staining in LysoTracker-positive endolysosomes from multiple cells within a field of view. A minimum of 77 cells per condition from two biological replicates were analyzed using Imaris 3D software. One-way ANOVA with Tukey's multiple comparison test was used for statistical analyses. ****$p < 0.0001$

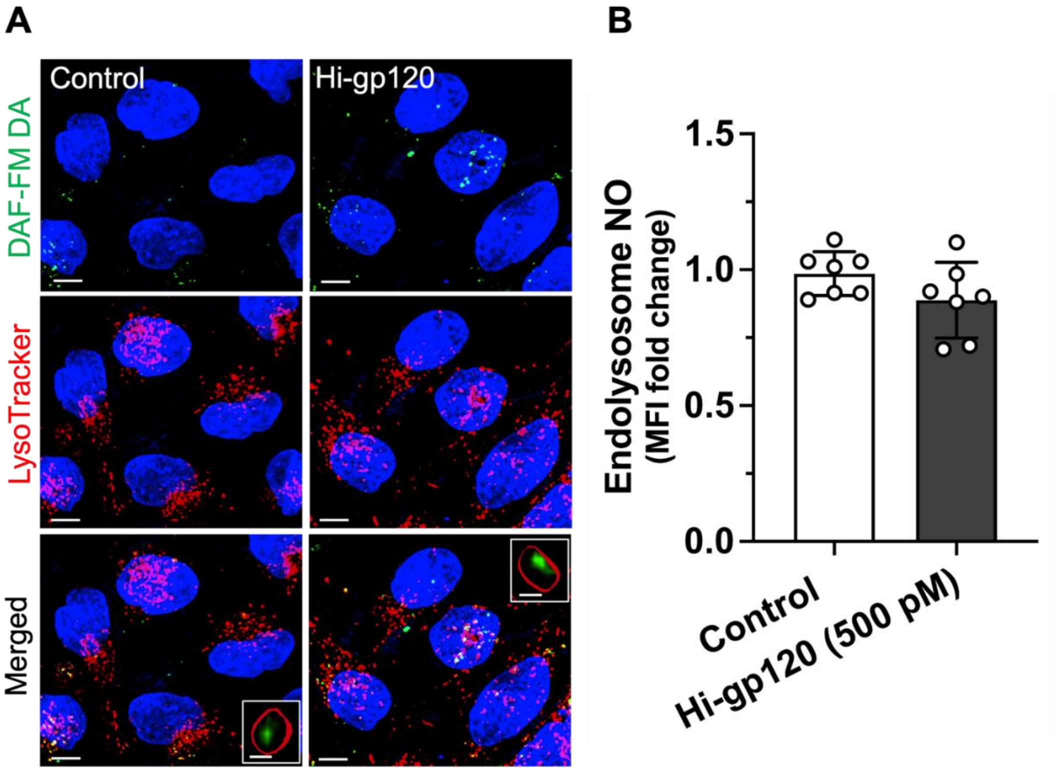
A
B
Control
Hi-gp120
DAF-FM DA
LysoTracker
Merged
Endolysosome NO
(MFI fold change)
1.5
1.0
0.5
0.0
Control
Hi-gp120 (500 pM)

Supplementary Figure S5. Heat-inactivated gp120 did not significantly affect levels of endolysosome nitric oxide. (A) Representative confocal images of SH-SY5Y cells incubated with water (control) and Hi-gp120 (500 pM) for 1 h and stained for nitric oxide (NO) with DAF-FM DA (green), endolysosomes (LysoTracker far red, red) and nuclei (Hoechst 33342, blue). Scale bars = 10 μm. Boxed inserts are magnifications of single composite endolysosomes containing NO. Scale bar = 1 μm. **(B)** Confocal Images were analyzed using Imaris 3D software and data were represented as fold changes in mean fluorescence intensity (MFI) of DAF-FM DA staining in LysoTracker-positive endolysosomes. Data were shown as mean ± SD, with individual data points (n = 7) included on each bar. Each data point (n) represents MFI of DAF-FM DA straining in LysoTracker-positive endolysosomes from multiple cells. A minimum of 77 cells per condition from two biological replicates were analyzed using Imaris 3D software. Two-tailed Student's *t*-test was used for statistical analyses.

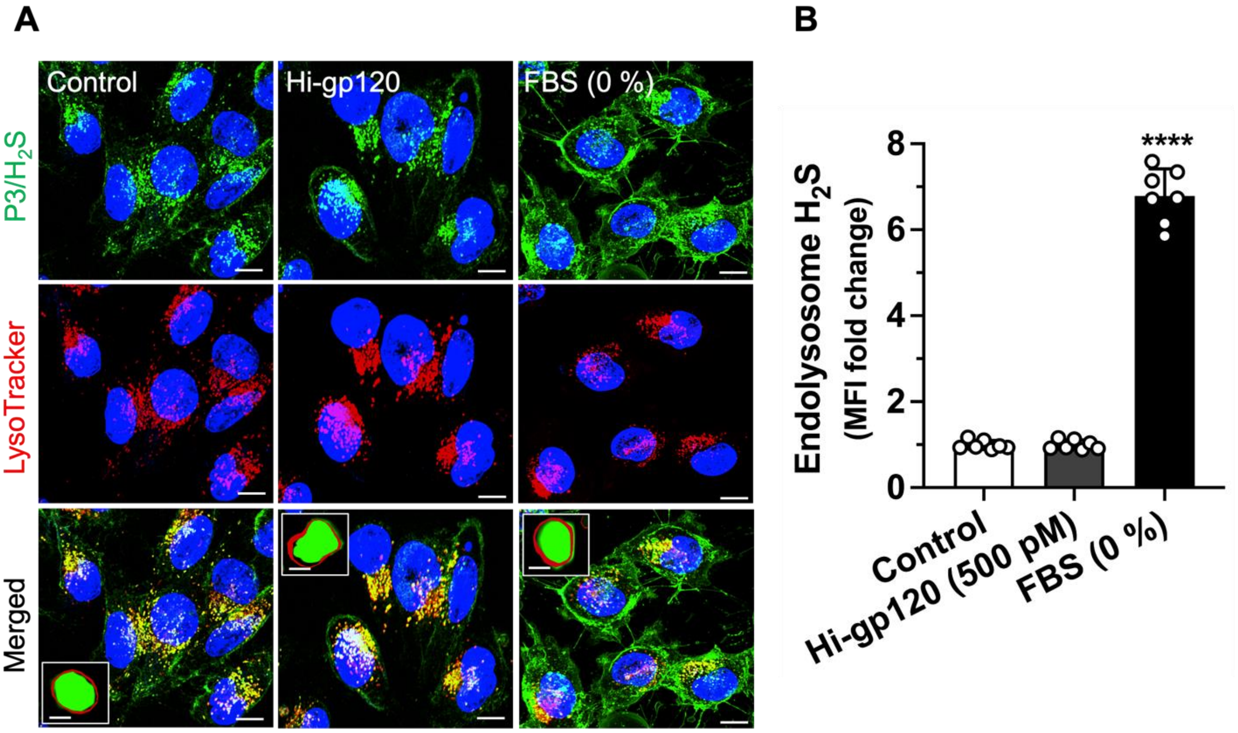
A
B
Control
Hi-gp120
FBS (0 %)
P3/H2S
LysoTracker
Merged
Endolysosome H2S
(MFI fold change)
8
6
4
2
0
****
Control
Hi-gp120 (500 pM)
FBS (0 %)

Supplementary Figure S6. Heat-inactivated gp120 did not significantly affect levels of endolysosome H_2S. (A) Representative confocal images of SH-SY5Y cells incubated for 1 h with water (control), Hi-gp120 (500 pM), and FBS-free medium (FBS 0 %) and stained for H_2S (P3 probe, green), endolysosomes (LysoTracker far red, red) and nuclei (Hoechst 33342, blue). Scale bars = 10 μm. Boxed inserts are magnifications of single composite endolysosomes containing H_2S. Scale bar = 1 μm. **(B)** Confocal Images were analyzed using Imaris 3D software and data were represented as fold changes in mean fluorescence intensity (MFI) of P3 in LysoTracker-positive endolysosomes. Data were presented as mean ± SD, with individual data points (n = 7) included on each bar. Each data point (n) represents MFI of P3 in LysoTracker-positive endolysosomes from multiple cells within a field of view. A minimum of 77 cells per condition from two biological replicates were analyzed using Imaris 3D software. One-way ANOVA with Tukey's multiple comparison test was used for statistical analyses. ****$p < 0.0001$

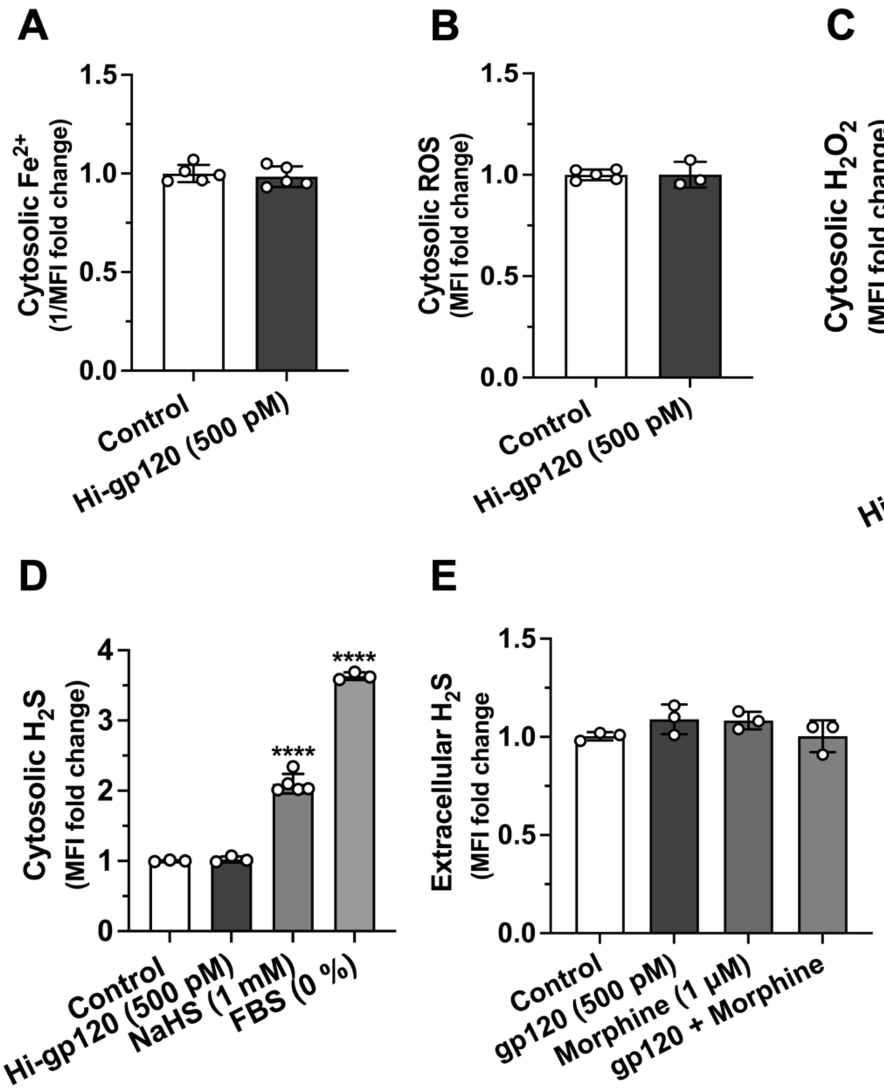
A
Cytosolic Fe2+
(1/MFI fold change)
Control
Hi-gp120 (500 pM)
B
Cytosolic ROS
(MFI fold change)
Control
Hi-gp120 (500 pM)
C
Cytosolic H2O2
(MFI fold change)
**
Control
Hi-gp120 (500 pM)
H2O2 (50 μM)
D
Cytosolic H2S
(MFI fold change)
****
****
Control
Hi-gp120 (500 pM)
NaHS (1 mM)
FBS (0 %)
E
Extracellular H2S
(MFI fold change
Control
gp120 (500 pM)
Morphine (1 μM)
gp120 + Morphine

Supplementary Figure S7. Heat-inactivated gp120 did not significantly affect the levels of cytosolic Fe^{2+}, ROS, H_2O_2, and H_2S. Levels of cytosolic Fe^{2+} were measured with the quenching dye PhenGreen FL, diacetate (PGFL, DA) and data were transformed and illustrated as the reciprocal of mean fluorescence intensity (1/MFI). Levels of cytosolic ROS were measured with CM-H_2DCFDA and levels of cytosolic H_2O_2 were measured with an H_2O_2 assay kit. Levels of cytosolic H_2S and culture medium (extracellular) H_2S were measured with SF7-AM; data were represented as fold changes in MFI. **(A-D)** Treatment of SH-SY5Y cells for 1 h with Hi-gp120 (500 pM) had no significant effect on levels of cytosolic Fe^{2+}, ROS, H_2O_2, and H_2S. H_2O_2 (50 μM) significantly increased levels of cytosolic H_2O_2. Levels of cytosolic H_2S were significantly increased by NaHS (H_2S donor) and serum-free media (0 % FBS). **(E)** 1 h treatment of cells with gp120 (500 pM) and morphine (1 μM) alone and in combination had no significant effect on levels of extracellular H_2S. Data were shown as mean ± SD, with individual data points (n = 3-6) included on each bar. One-way ANOVA with Tukey's multiple comparison test or two-tailed Student's *t*-test was used for data analysis. $^{**}p< 0.01$, $^{****}p< 0.0001$

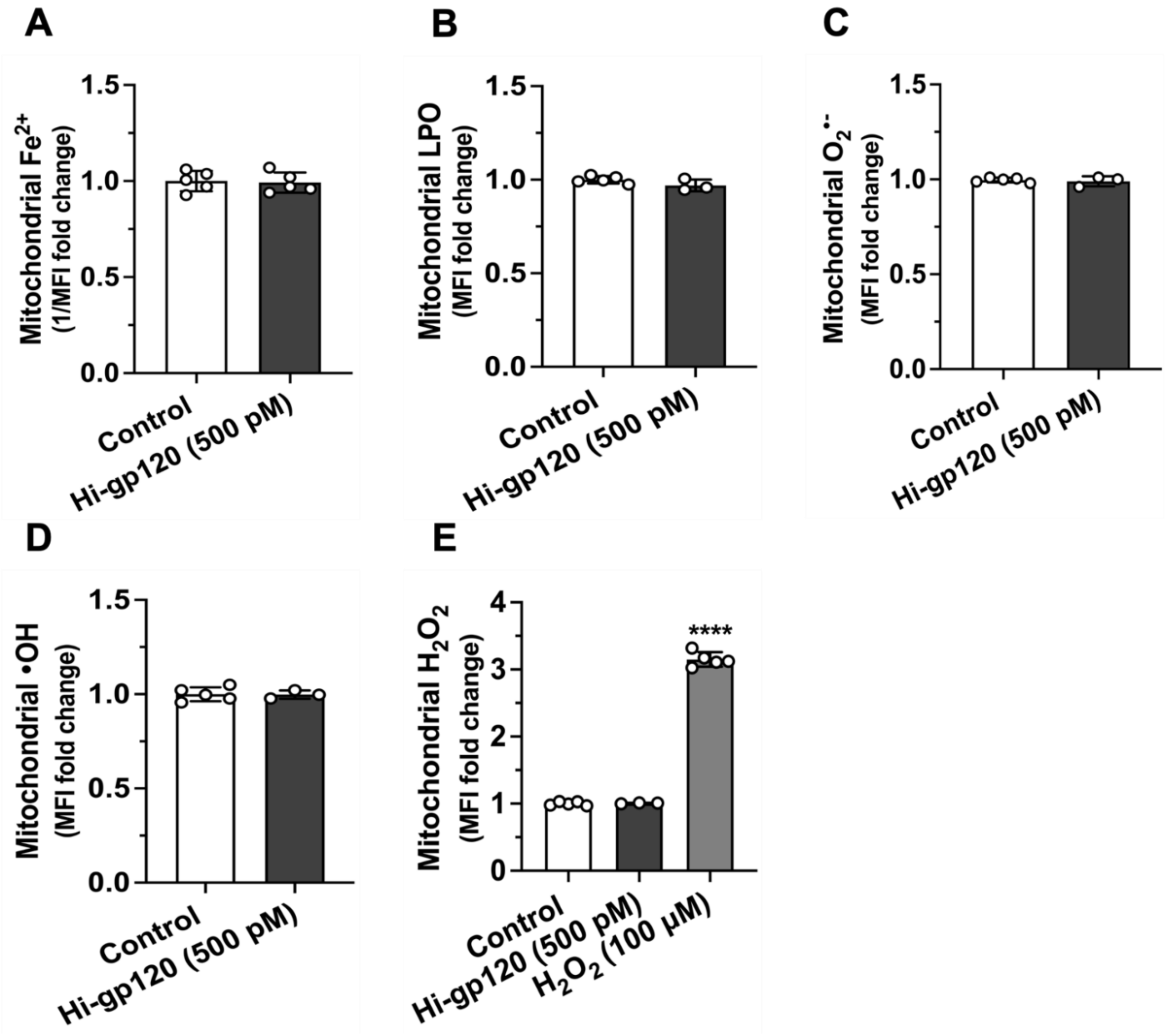
A
Mitochondrial Fe2+
(1/MFI fold change)
1.5
1.0
0.5
0.0
Control
Hi-gp120 (500 pM)
B
Mitochondrial LPO
(MFI fold change)
1.5
1.0
0.5
0.0
Control
Hi-gp120 (500 pM)
C
Mitochondrial O2•−
(MFI fold change)
1.5
1.0
0.5
0.0
Control
Hi-gp120 (500 pM)
D
Mitochondrial •OH
(MFI fold change)
1.5
1.0
0.5
0.0
Control
Hi-gp120 (500 pM)
E
Mitochondrial H2O2
(MFI fold change)
4
3
2
1
0
****
Control
Hi-gp120 (500 pM)
H2O2 (100 µM)

Supplementary Figure S8. Heat-inactivated gp120 did not significantly affect the levels of mitochondrial Fe^{2+}, LPO, $O_2^{\bullet-}$, $^{\bullet}OH$, and H_2O_2. Levels of mitochondrial Fe^{2+} were measured with the quenching dye RDA and data were transformed and illustrated as the reciprocal of mean fluorescence intensity (1/MFI). Levels of mitochondrial LPO, $O_2^{\bullet-}$, $^{\bullet}OH$, and H_2O_2 were measured using MitoPerOx, MitoSOX, OH580 $^{\bullet}OH$ kit, and MitoPY1, respectively; data were collected using flow cytometry and represented as fold changes in MFI. **(A-E)** Hi-gp120 (500 pM) did not significantly affect levels of mitochondrial Fe^{2+}, LPO, $O_2^{\bullet-}$, $^{\bullet}OH$, and H_2O_2. Data were shown as mean ± SD, with individual data points (n = 3-6) included on each bar. Two-tailed Student's *t*-test or one-way ANOVA Tukey's multiple comparison test was used for data analysis. ****$p < 0.0001$

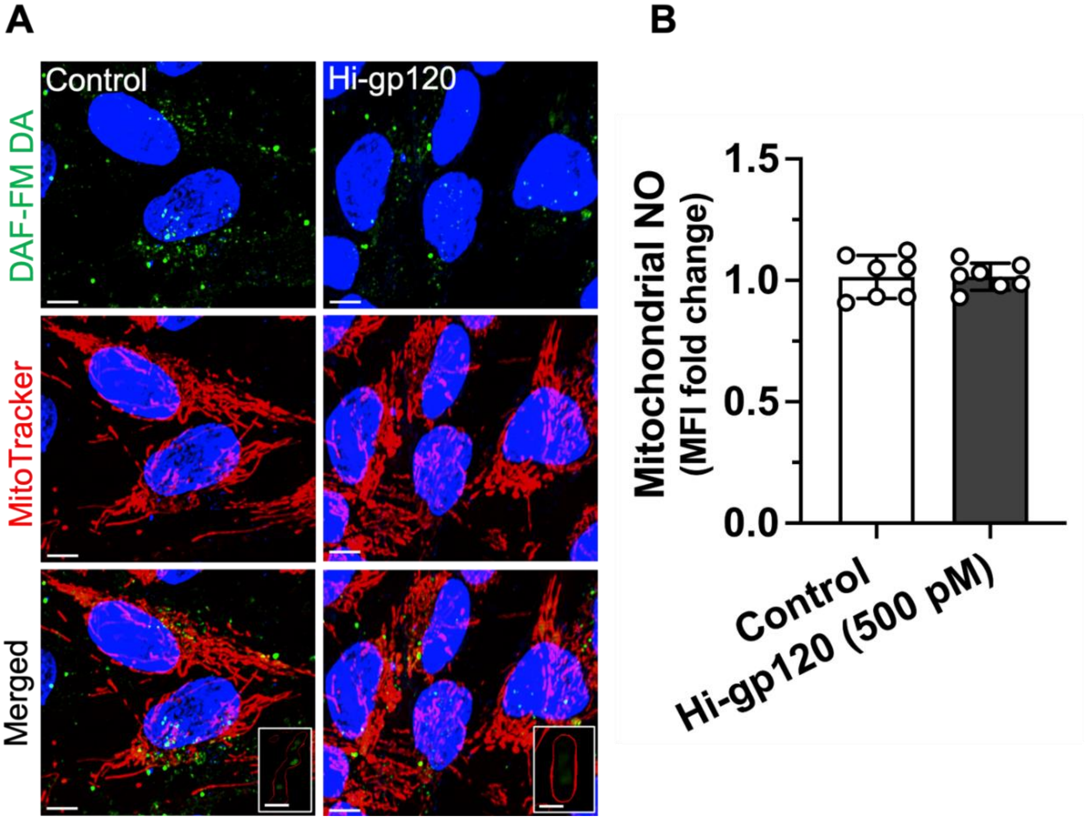
A
B
Control
Hi-gp120
DAF-FM DA
MitoTracker
Merged
Mitochondrial NO
(MFI fold change)
1.5
1.0
0.5
0.0
Control
Hi-gp120 (500 pM)

Supplementary Figure S9. Heat-inactivated gp120 did not significantly affect mitochondrial NO levels. Representative confocal images of SH-SY5Y cells incubated for 1 h with water (control) and Hi-gp120 (500 pM) and stained for NO with DAF-FM DA, (green), mitochondria (MitoTracker Red, red) and nuclei (Hoechst 33342, blue). Scale bars = 10 μm. Boxed inserts are magnified mitochondria containing NO. Scale bar = 1 μm. **(B)** Confocal images were analyzed using Imaris 3D software and data were represented as fold changes of mean fluorescence intensity (MFI) inside MitoTracker-positive mitochondria. Data were presented as mean ± SD, with individual data points (n = 7) illustrated in each bar. Each data point (n) represents MFI of DAF-FM DA staining inside mitochondria from multiple cells within a field of view. A minimum of 61 cells per condition from two biological replicates were analyzed using Imaris 3D software. Two-tailed Student's *t*-test was used to analyze the data.

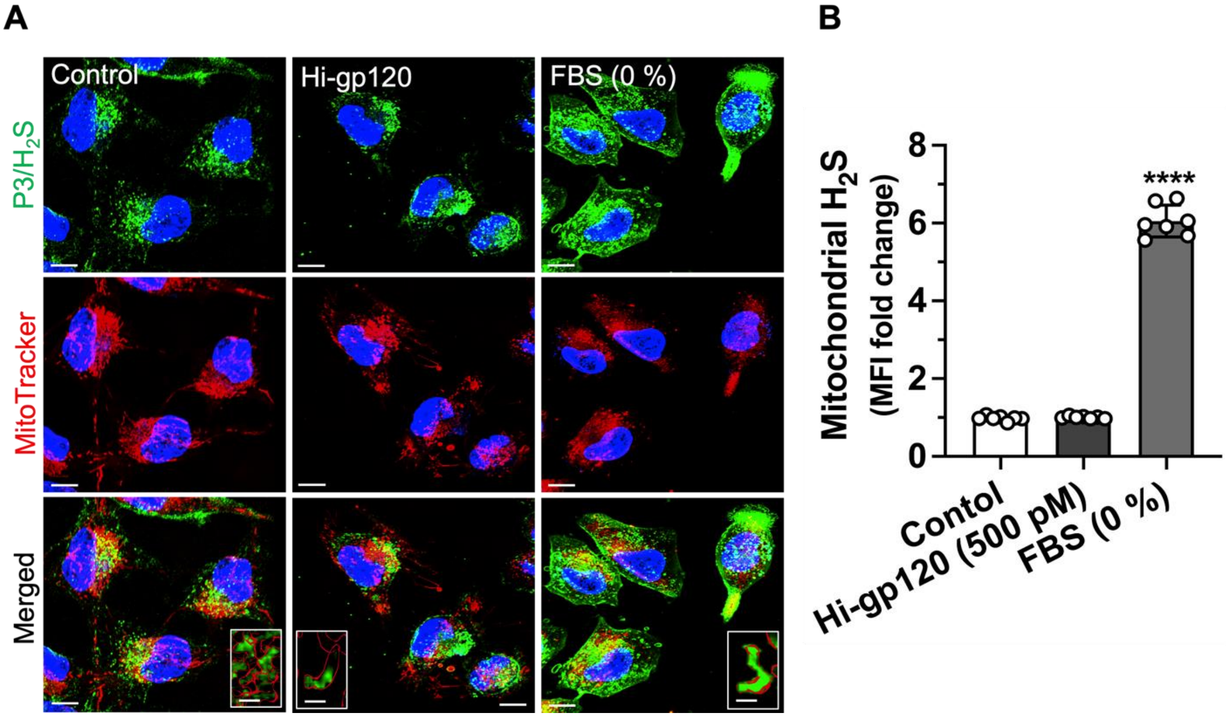
A
Control
Hi-gp120
FBS (0 %)
P3/H2S
MitoTracker
Merged
B
Mitochondrial H2S
(MFI fold change)
8
6
4
2
0
****
Contol
Hi-gp120 (500 pM)
FBS (0 %)

Supplementary Figure S10. Heat-inactivated gp120 did not significantly affect the levels of mitochondrial H_2S. (A) Representative confocal images of SH-SY5Y cells incubated for 1 h with water (control), Hi-gp120 (500 pM), and for 2 h in FBS-free medium (0% FBS) and stained for H_2S with P3 probe (green), mitochondria (MitoTracker red, red) and nuclei (Hoechst 33342, blue). Scale bars = 10 µm. Boxed inserts are magnified mitochondria containing H_2S. Scale bar = 1 µm. **(B)** Confocal Images were analyzed using Imaris 3D software and data were represented as fold changes in mean fluorescence intensity (MFI) inside MitoTracker-positive mitochondria. Hi-gp120 did not significantly affect levels of mitochondrial H_2S. FBS-depleted medium (0% FBS) significantly increased levels of mitochondrial H_2S. Bars (mean ± SD) and individual data points (n = 7) were illustrated. Each data point (n) represents MFI of P3 in mitochondria from multiple cells within a field of view. A minimum of 50 cells per condition from two biological replicates were analyzed using Imaris 3D software. One-way ANOVA with Tukey's multiple comparison test was used for statistical analyses. ****$p< 0.0001$

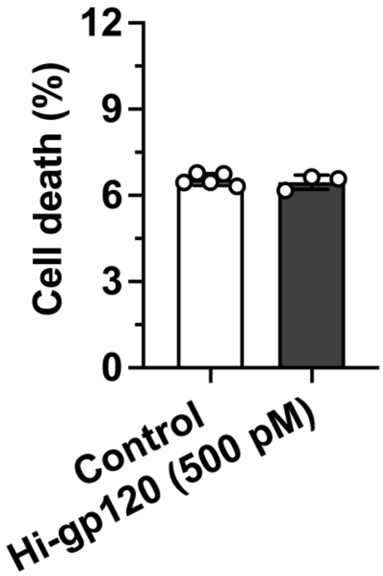
12
9
6
3
0
Cell death (%)
Control
Hi-gp120 (500 pM)

Supplementary Figure S11. Heat-inactivated gp120 did not significantly increase cell death. SH-SY5Y cells were incubated with Hi-gp120 (500 pM) for 24 h and stained with propidium iodide (PI). Data were collected using flow cytometry and presented as percent cell death. Hi-gp120 did not significantly affect cell death compared to controls. Data were shown as mean ± SD, with individual data points (n = 3-5) included on each bar. Two-tailed Student's *t*-test was used for statistical analyses.

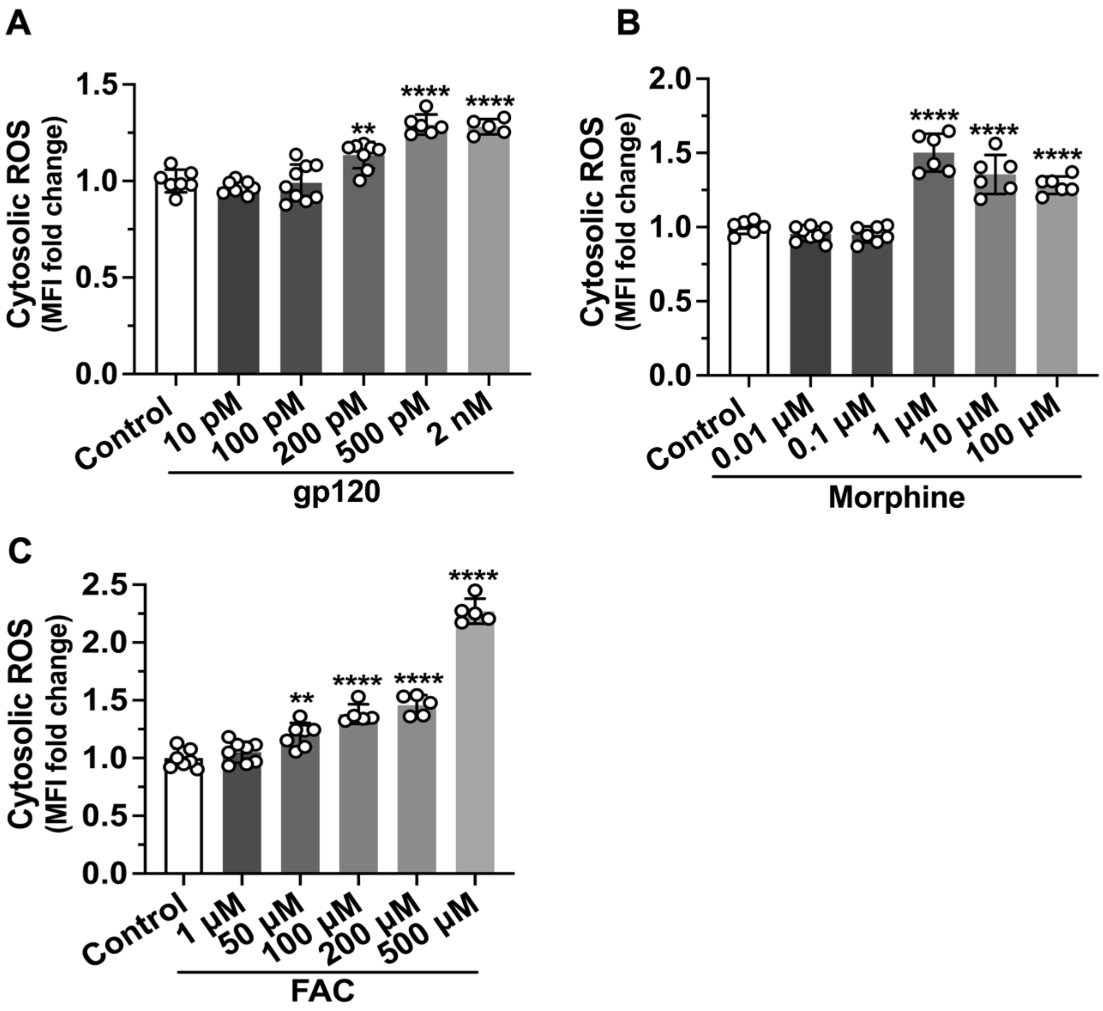

A
Cytosolic ROS
(MFI fold change)
1.5
1.0
0.5
0.0
**
****
****
Control
10 pM
100 pM
200 pM
500 pM
2 nM
gp120
B
Cytosolic ROS
(MFI fold change)
2.0
1.5
1.0
0.5
0.0
****
****
****
Control
0.01 μM
0.1 μM
1 μM
10 μM
100 μM
Morphine
C
Cytosolic ROS
(MFI fold change)
2.5
2.0
1.5
1.0
0.5
0.0
**
****
****
****
Control
1 μM
50 μM
100 μM
200 μM
500 μM
FAC

Supplementary Figure S12. Concentration-dependent effects of gp120, morphine, and FAC on levels of cytosolic ROS. Levels of cytosolic ROS were measured with CM-H_2DCFDA. **(A-C)** SH-SY5Y cells were treated for 1 h with different concentrations of gp120 (10 pM to 2 nM), morphine (0.01 μM to 100 μM), and FAC (1 μM to 500 μM). Data were presented as mean ± SD, with individual data points (n = 5-9) included on each bar. One-way ANOVA with Tukey's multiple comparison test was used for statistical analyses. $^{**}p < 0.01$, $^{****}p < 0.0001$
